# Efficacy and safety of 12 immunosuppressive agents for idiopathic membranous nephropathy in adults: A pairwise and network meta-analysis

**DOI:** 10.3389/fphar.2022.917532

**Published:** 2022-07-25

**Authors:** Jiarong Liu, Xiang Li, Tianlun Huang, Gaosi Xu

**Affiliations:** Department of Nephrology, The Second Affiliated Hospital of Nanchang University, Nanchang, China

**Keywords:** idiopathic membranous nephropathy, immunosuppressant, network meta-analysis, adverse effects, therapies

## Abstract

**Background:** Immunosuppressants have been applied in the remedy of idiopathic membranous nephropathy (IMN) extensively. Nevertheless, the efficacy and safety of immunosuppressants do not have final conclusion. Thus, a pairwise and network meta-analysis (NMA) was carried out to seek the most recommended therapeutic schedule for patients with IMN.

**Methods:** Randomized controlled trials (RCTs) including cyclophosphamide (CTX), mycophenolate mofetil (MMF), tacrolimus-combined mycophenolate mofetil (TAC + MMF), cyclosporine (CsA), tacrolimus (TAC), leflunomide (LEF), chlorambucil (CH), azathioprine (AZA), adrenocorticotropic hormone (ACTH), non-immunosuppressive therapies (CON), steroids (STE), mizoribine (MZB), and rituximab (RIT) for patients with IMN were checked. Risk ratios (RRs) and standard mean difference (SMD) were reckoned to assess dichotomous variable quantities and continuous variable quantities, respectively. Total remission (TR) and 24-h urine total protein (24-h UTP) were compared using pairwise and NMA. Then interventions were ranked on the basis of the surface under the cumulative ranking curve (SUCRA).

**Results:** Our study finally included 51 RCTs and 12 different immunosuppressants. Compared with the CON group, most regimens demonstrated better therapeutic effect in TR, with RR of 2.1 (95% CI) (1.5–2.9) for TAC, 1.9 (1.3–2.8) for RIT, 2.5 (1.2–5.2) for TAC + MMF, 1.9 (1.4–2.7) for CH, 1.8 (1.4–2.4) for CTX, 2.2 (1.0–4.7) for ACTH, 1.6 (1.2–2.1) for CsA, 1.6 (1.0–2.5) for LEF, and 1.6 (1.1–2.2) for MMF. In terms of 24-h UTP, TAC (SMD, −2.3 (95% CI −3.5 to −1.1)), CTX (SMD, −1.7 (95% CI −2.8 to −0.59)), RIT (SMD, −1.8 (95% CI −3.5 to −0.11)), CH (SMD, −2.4 (95% CI −4.3 to −0.49)), AZA (SMD, −−4.2 (95% CI −7.7 to −0.68)), and CsA (SMD, −1.7 (95% CI −3 to −0.49)) were significantly superior than the CON group. As for adverse effects (AEs), infections, nausea, emesia, myelosuppression, and glucose intolerance were the collective adverse events for most immunosuppressants.

**Conclusion:** This study indicates that TAC + MMF performed the best in terms of TR, and TAC shows the best effectiveness on 24-h UTP compared with other regimens. On the contrary, there seems to be little advantage on STE alone, LEF, AZA, and MZB in treating patients with IMN compared with CON.

**Systematic Review Registration:** [https://www.crd.york.ac.uk/prospero/], identifier [CRD42021287013]

## 1 Introduction

Idiopathic membranous nephropathy (IMN) is the main cause of adult nephrotic syndrome, accounting for about 20% of nephrotic syndrome (NS). Among patients with IMN, the elderly accounts for up to 50% (Haas et al., 1997). IMN is a glomerular disease characterized by the deposition of immune complexes on the epithelial side of the glomerular capillary wall and diffuse thickening of the basement membrane. About 31.7% of patients with IMN spontaneously relieved within 2 years after conservative treatment, while about 30%–40% of patients had progressive decline of renal functions and finally developed into end-stage renal disease (ESRD) (Polanco et al., 2010). These variable processes of IMN have caused great difficulties for physicians when deciding the optimum treatment regimen.

There are many treatment methods for IMN, from the general treatment to immunosuppressive therapy, calcineurin inhibitors, adrenocorticotropic hormone therapy, monoclonal antibody therapy, and anticoagulant drugs. Among these treatment regimens, immunosuppressive therapy is highly recommended for patients with persistent heavy proteinuria, which can not only reduce the recurrence of disease but also slow down the progress to ESDR (Hofstra et al., 2013). It has been more than 30 years since immunosuppressants were used to treat IMN for the first time (Polanco et al., 2010). Since then, various immunosuppressive therapies have been put forward. However, the curative effect and the adverse reaction of different immunosuppressants remain unknown (van de Logt et al., 2016). Understanding the mechanism and efficacy of different immunosuppressants, making individualized treatment, and maximizing the benefit of patients according to the specific conditions of patients are the current clinical problems. However, it is difficult to draw a clear conclusion through traditional meta-analysis and RCTs. Therefore, aiming to provide a clinical reference, this study uses the method of network meta-analysis (NMA) to compare the effectiveness and safety of immunosuppressive agents combining direct and indirect results.

NMA is a more valuable means than traditional meta-analysis, whose characteristics are not only multivariate and multilevel comprehensive analysis but also ranking multiple interventions simultaneously by combining direct and indirect comparison results (Salanti, 2012). It could increase accuracy and reliability of final conclusion through this tool (Tonin et al., 2017). Therefore, a pairwise and network meta-analysis to rank effectiveness and safety of different immunosuppressants for patients with IMN was performed.

## 2 Materials and methods

Our study was conducted on the basis of PRISMA (referring to the Preferred Reporting Items for Systematic Review and Meta-analysis) checklists and guidelines (Page et al., 2021). It is shown as a supplementary file about the PRISMA checklist (see Supplementary File S1). Also, our study has been registered in the International Prospective Register of Systematic Reviews (PROSPERO: CRD42021287013).

### 2.1 Data sources and searches

We extensively retrieved databases of PubMed, Cochrane Library, Web of Science, clinicaltrials.gov, SinoMed, Chinese Biomedicine, China National Knowledge Infrastructure, WanFang, and VIP Information from beginning to July 2021 for RCTs observing clinical efficacy of any different immunosuppressive agents for patients with IMN. There are no special conditions attached on the language, years of publication, or methods of blinding. The retrieval strategies were carried out using a combination of MeSH terms and free words. The detailed searching strategy was as follows: [(Membranous Glomerulonephritides) OR (Membranous Glomerulonephritis) OR (Membranous Glomerulopathy) OR (Membranous Nephropathy) OR (Extramembranous Glomerulopathy) OR (Idiopathic Membranous Glomerulonephritis) OR (Membranous Glomerulonephropath) OR (Idiopathic Membranous Nephropathy) OR (Idiopathic Membranous Glomerulonephritides)] AND [(Tacrolimus) OR (Cyclophosphamide) OR (Rituximab) OR (Cyclosporin) OR (Mycophenolate mofetil) OR (Steroids) OR (Adrenocorticotropic hormone) OR (Azathioprine) OR (Chlorambucil) OR (Leflunomide) OR (Mizoribine)]. In addition, we manually screened the literature list to prevent the omission of appropriate literatures as well.

### 2.2 Selection criteria

The selection criteria for included publications were as follows: 1) the type of study should be randomized controlled trials (RCTs) (see Supplementary Table S1 for specific selection criteria); 2) patients in the studies must have been conformed as IMN by renal biopsy and have proteinuria at the level of nephrotic syndrome (24-h urine total protein >3.5 g), and all patients included in the study must be treated for more than 6 months; and 3) each study should report the number of patients with total remission (TR) or 24-h urine total protein (24-h UTP). TR was defined as either complete or partial remission (CR or PR), and CR was defined as 24-h urine total protein <0.3 g, authenticated by two text results with an interval of more than 1 week. PR was defined as 24-h urine total protein <3.5 g and a decrease of half or more from the crest value, authenticated by two text results with an interval of more than 1 week (Floege et al., 2019). 4) Interventions for studies should include CsA (cyclosporine), CTX (cyclophosphamide), LEF (leflunomide), MMF (mycophenolate mofetil), ACTH (adrenocorticotropic hormone), TAC (tacrolimus), AZA (azathioprine), CH (chlorambucil), CON (non-immunosuppressive therapies), MZB (mizoribine), RIT (rituximab), STE (steroids), and TAC + MMF (tacrolimus-combined mycophenolate mofetil). 5) Especially, in terms of the treatment of rituximab, we only selected randomized clinical trials that receive intravenous rituximab (two infusions, 1,000 mg each or four infusions, 375 mg each). As for concomitant medication, those patients assigned to the TAC + MMF group received both TAC (an initial dose of 2 mg twice daily titrated to achieve whole blood levels of 5–12 ng/ml) and MMF (500 mg twice daily titrated to achieve blood mycophenolic acid (MPA) levels of 1.5–3.0 mg/L). Publications conforming to the following criteria were excluded: 1) study participants were not adults (younger than 16 years); 2) the study was not designed to observe the efficacy and safety of different medications for patients with IMN; 3) the treatment had not to be first-line or patients had received immunotherapies before the study; 4) patients with secondary membranous nephropathy, IMN after kidney transplantation, or atypical membranous nephropathy; and 5) drugs included in the publication were not involved in our study, or publications compared the same drug in terms of administration route or dosage.

### 2.3 Data extraction and quality evaluation

We used EndNote software to manage the retrieved literature. After screening the title and abstract, the article meeting the inclusion criteria was obtained for evaluation and data extraction. In addition, two reviewers (JRL and TLH) extracted data independently through Microsoft Excel. Different opinions in the process of data extraction shall be solved by the third reviewer (GSX). The contents of data extraction included basic characteristics of the included literature (country, publication year, and first author), subjects for study information (mean age, sex ratio, sample size, basal blood pressure, and baseline of 24-h UTP), interventions (different immunosuppressive regimens, course of treatment, and period of follow-up), and reported outcomes (TR, 24-h UTP, and AEs). For information that cannot be obtained directly, we make great efforts to contact the author *via* email. The two reviewers (JRL and TLH) assessed the risk of bias for all studies independently according to the Cochrane Risk of Bias tool [Cochrane Handbook for Systematic Reviews of Interventions, version 5.4.0] (Higgins et al., 2011). Each domain can be evaluated as high, low, or unclear risk for included studies. Disagreements will be resolved by the third reviewer (GSX).

### 2.4 Statistical analysis

The number of patients with TR, 24-h UTP, and adverse effects were extracted from publications. Then our network meta-analysis within a frequentist framework adopted the random-effects model (Greco et al., 2015). We calculated standard mean difference (SMD) with 95% confidence intervals (95% CI) for continuous variables and risk ratios (RR) with 95% confidence intervals (95% CI) for dichotomous variables to describe the effect sizes. STATA 14.0 software was applied to perform the conventional pairwise meta-analysis for ascertaining the effects of immunosuppressive agents. Before carrying out our statistical analysis, we installed the STATA 14.0 (“mvmeta” and “network” packages) to draw the network diagram, make league tables, and assess for publication bias and R 3.5.1 (“ggplot2″ and “gemtc” packages) to draw forest plots and regression analysis. The R 3.5.1 was applied for a Bayesian frame structure, while STATA 14.0 was employed for a frequentist framework. We generated 1,000,000 simulations for each of the two sets of different initial values and discarded the first 50,000 simulations as the burn-in period. Then the convergence and density diagrams were examined by using Brooks–Gelman–Rubin diagnostic and trace plots. If zero is not included in the range of the 95% CI of SMD, or one is not included in the range of the 95% CI of RR, the difference between the two comparison groups is considered to be statistically significant. The probabilities of being at each possible rank for each therapeutic regimen were calculated. The therapeutic regimes were concluded and reported according to surface under the cumulative ranking curve (SUCRA) and mean ranks. SUCRA, as a percentage, is interpreted as the probability of a therapeutic schedule to become the most effective on the outcome, which is infinitely close to one when the treatment is considered to be the best and infinitely close to zero when it is regarded to be the worst (Salanti et al., 2011). Higher SUCRA values manifested higher treatment grades. There is always some heterogeneity among the included studies, which is inevitable. Hence, to evaluate the consistency of NMA, we adopted the “design-by-treatment” model (Higgins et al., 2012) for global assessment and the node-splitting method (Veroniki et al., 2013) for local assessment to estimate statistical consistency within every closed loop. The node-splitting method split the same comparison into direct and indirect comparisons and used *p*-values to assess the difference between them (Yu-Kang, 2016). It is considered that the heterogeneity is not significant if *p* > 0.05 in direct and indirect comparisons. Then we would adopt the consistency model for following statistical analysis. Pairwise and network heterogeneity were evaluated using *I* ^*2*^, and *I* ^*2*^ more than 50% indicated significant heterogeneity. However, using inconsistency or consistency model, the *I* ^*2*^ values of TR and 24-h UTP were less than 6%, indicating low heterogeneity overall (see Supplementary Table S2). In addition, meta-regression and sensitivity analyses were conducted to investigate the potential source of heterogeneity by R and STATA software.

### 2.5 General classification of drugs

Combining the SUCRA ranking of TR and 24-h UTP, we will roughly divide these immunosuppressants into four groups: significant effect group (SUCRA of both two are greater than 60%), moderate effect group (SUCRA of both two are between 40%–60%), low effect group (SUCRA of both two are lower than 40%), and very low effect group (SUCRA of both two are ranked at the bottom). The horizontal axis represents the SUCRA of different treatments on total remission, whereas the longitudinal axis represents the SUCRA of different treatments on 24-h UTP.

## 3 Results

### 3.1 Selection and identification of studies

**FIGURE 1:**
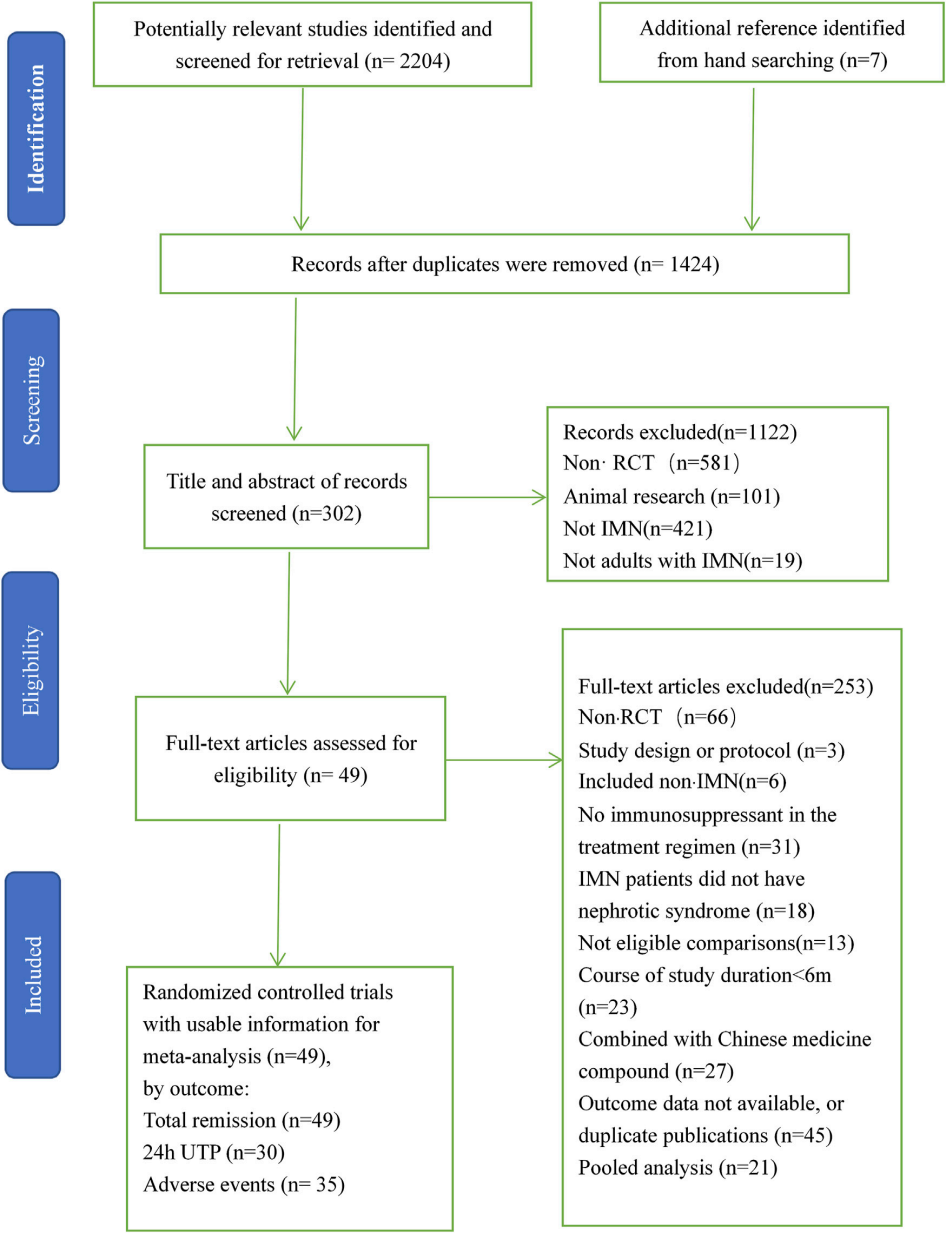
Flow chart of literature search and selection. IMN, idiopathic membranous nephropathy; RCTs, randomized clinical trials.

Altogether 2,213 studies were recognized, among which includes 787 reduplicated studies, and then 304 articles were identified after excluding 1,122 studies because of non-RCT, animal research, not IMN, or not adults with IMN by means of the titles, keywords, and abstracts. A total of 253 articles were removed after we skimmed 304 full-text because of substandard study design, no immunosuppressant in the treatment regimen, study object, study duration, or outcomes. Ultimately, 51 RCTs (including 49 two-armed RCTs and two three-armed RCTs) (15–65) including 2,830 patients were available for pairwise and network meta-analysis. These RCTs observed 12 different immunosuppressants, including CTX (cyclophosphamide), MZB (mizoribine), RIT (rituximab), STE (steroids), AZA (azathioprine), ACTH (adrenocorticotropic hormone), CH (chlorambucil), CsA (cyclosporine), LEF (leflunomide), MMF (mycophenolate mofetil), TAC (tacrolimus), and TAC + MMF (tacrolimus-combined mycophenolate mofetil) for patients with IMN. Figure 1 is the specific flow diagram.

### 3.2 Included study characteristics

The mean treatment duration was 9.63 months (range: 6–36 months). Of these 51 RCTs, CTX was utilized in 28 RCTs and 743 patients with highest frequency (28 RCTs, 743 patients), CON (18 RCTs, 516 patients), TAC (12 RCTs, 316 patients), CsA (14 RCTs, 325 patients), CH (7 RCTs, 230 patients), MMF (7 RCTs, 134 patients), RIT (5 RCTs, 198 patients), STE (5 RCTs, 227 patients), and LEF (4 RCTs, 81 patients); AZA, MZB, ACTH, and TAC + MMF were all applied in one RCT with 13, 11, 16, and 20 patients, respectively. All 51 RCTs reported the detailed information about TR (including CR and PR), 32 of which reported the baseline of 24-h UTP and later 24-h UTP after receiving relevant treatment. Meanwhile, 40 RCTs mentioned different adverse effects both in the treatment group and the control group. The characteristics of the 51 included RCTs are shown in Supplementary Table S3. A random grouping method is mentioned among all eligible studies except three RCTs. What’s more, 24 RCTs (47%) described specific randomization methods, of which 19 RCTs used randomization number tables, four RCTs used stratified random sampling, and one RCT used block randomization, all of them were classified as “low risk” in random sequence generation. All RCTs presenting complete data and no selecting outcomes to report were regarded as “low risk” of bias in complete outcome assessment and a selective reporting domain. Nevertheless, due to lack of sufficient information, most RCTs were considered as “unclear risk” in terms of performance bias, detection bias, and other biases. The risks of biases of the eligible studies are shown in Supplementary File S2.

### 3.3 Network structure, consistency, and heterogeneity

**FIGURE 2:**
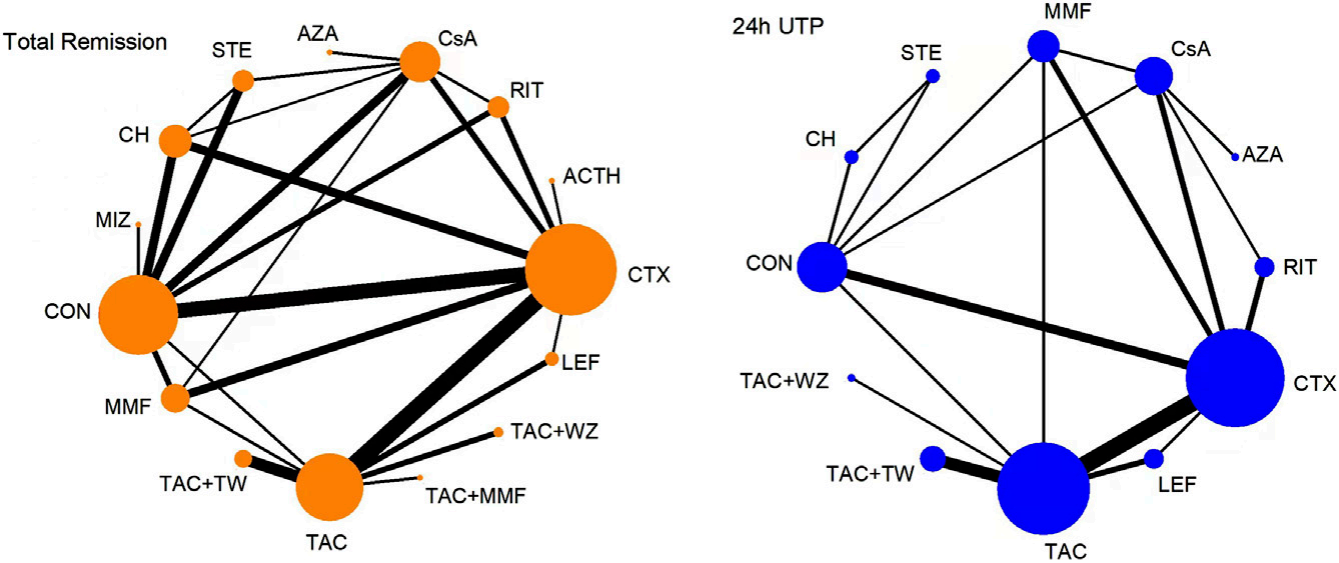
Network meta-analysis of eligible comparisons for total remission and 24-h UTP. The width of the lines represents the number of each pairwise comparison. The size of each node is proportional to the number of randomly assigned participants (ie., sample size). ACTH, adrenocorticotropic hormone; AZA, azathioprine; CH, chlorambucil; CON, non-immunosuppressive therapies (the control group); CsA, cyclosporine; CTX, cyclophosphamide; LEF, leflunomide; MMF, mycophenolate mofetil; MZB, mizoribine; RIT, rituximab; STE, steroids; TAC, tacrolimus; and TAC + MMF, tacrolimus-combined mycophenolate mofetil.

Figure 2 shows a network plot of treatment comparisons, the number of interventions was 15 for TR and 12 for 24-h UTP. The size of the nodes correlated with the intervention’s sample size. Also, the straight line whose thickness associated with the test number of direct comparison was used to connect different treatment regimens. As shown in Figure 2, the sample size and comparison times of different interventions were different.

The diagnostic and trace plots demonstrated that the convergence of this NMA was satisfactory. As presented in Supplementary File S3, the node-splitting methods were used to conduct consistency analysis, and all the *p* values were greater than 0.05, except the comparison between CTX and CsA for TR; MMF and CON, LEF and CTX, and TAC and LEF for 24-h UTP, which indicated that our work had high consistency and reliability. In the heterogeneity analysis (Supplementary File S4), significant heterogeneity could be found in the comparison of CTX and CH and RIT and CsA for TR, MMF and CON, STE and CON, and LEF and CTX for 24-h UTP. That was why we chose the random-effects model to conduct network meta-analysis and performed meta-regression and sensitivity analyses to look for the sources of heterogeneity.

### 3.4 Pairwise meta-analysis

The results of the pairwise meta-analysis among 12 immunosuppressive agents are shown in Supplementary File S5.

### 3.5 Network meta-analysis

#### 3.5.1 TR

TR was reported in 51 publications involving 2,830 patients. A total of 13 interventions were included: CTX (28 trials, 743 patients), CON (18, 516), TAC (12, 316), CsA (14, 325), CH (7, 230), MMF (7, 134), RIT (5, 198), STE (5, 227), LEF (4, 81), AZA (Haas et al., 1997; Higgins et al., 2012), MIZ (Haas et al., 1997; Greco et al., 2015), ACTH (Haas et al., 1997; Branten et al., 1998), and TAC + MMF (Haas et al., 1997; Chen et al., 2010). The network plot is presented in Figure 2.

**FIGURE 3:**
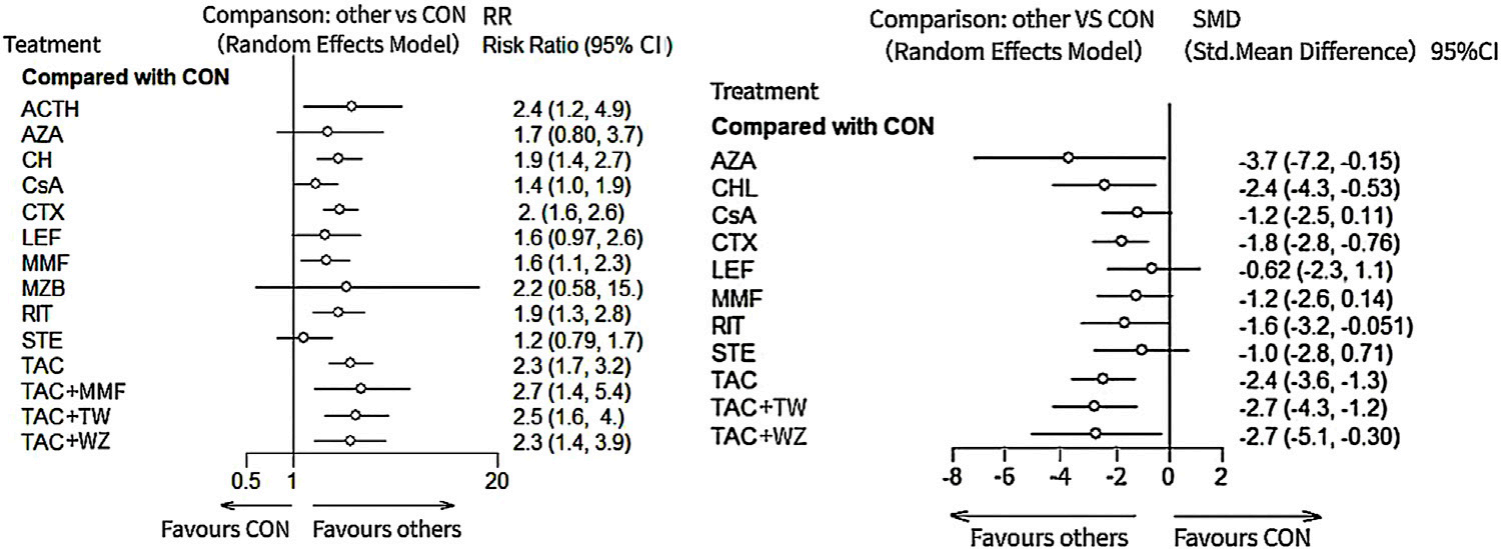
Result of network meta-analysis for total remission and 24-h urine total protein. ACTH, adrenocorticotropic hormone; AZA, azathioprine; CH, chlorambucil; CON, non-immunosuppressive therapies (the control group); CsA, cyclosporine; CTX, cyclophosphamide; LEF, leflunomide; MMF, mycophenolate mofetil; MZB, mizoribine; RIT, rituximab; STE, steroids; TAC, tacrolimus; and TAC + MMF, tacrolimus combined-mycophenolate mofetil.

**FIGURE 4:**
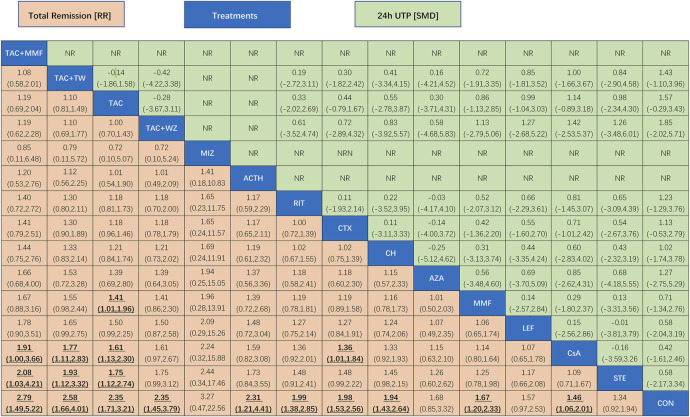
League table of all comparisons of total remission and 24-h UTP. Data are RRs (95% CI) for total remission (lower-left quadrant) and MDs (95% CI) for 24-h UTP (upper-right quadrant) in the column-defining treatment compared with the row-defining treatment. RRs higher than one favor the column-defining treatment and MDs lower than zero favor the row-defining treatment. Significant results are in bold and underscored. ACTH, adrenocorticotropic hormone; AZA, azathioprine; CH, chlorambucil; CON, non-immunosuppressive therapies (the control group); CsA, cyclosporine; CTX, cyclophosphamide; LEF, leflunomide; MMF, mycophenolate mofetil; MZB, mizoribine; RIT, rituximab; STE, steroids; TAC, tacrolimus; and TAC + MMF, tacrolimus combined mycophenolate mofetil.

As illustrated in Figure 3 and Figure 4, Compared with CON, all the remedies demonstrated better therapeutic effect in TR, except for MZB, AZA, and STE, with risk ratio (RRs) of 2.1 (95% CI) (1.5–2.9) for TAC, 1.9 (1.3–2.8) for RIT, 2.5 (1.2–5.2) for TAC + MMF, 1.9 (1.4–2.7) CH, 1.8 (1.4–2.4) for CTX, 2.2 (1.0–4.7) for ACTH, 1.6 (1.2–2.1) for CsA, 1.6 (1.1–2.2) for MMF, and 1.6 (1.2–2.1) for CsA.

**FIGURE 5:**
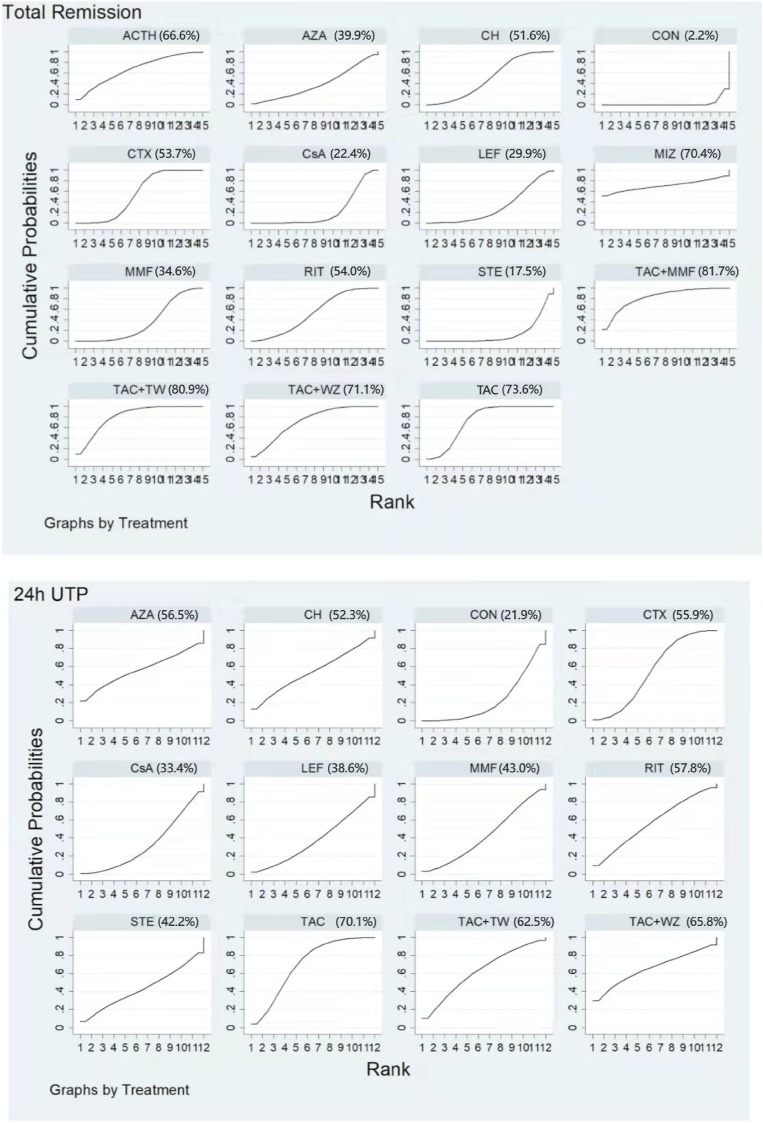
Rankings of SUCRA for all treatments. ACTH, adrenocorticotropic hormone; AZA, azathioprine; CH, chlorambucil; CON, non-immunosuppressive therapies (the control group); CsA, cyclosporine; CTX, cyclophosphamide; LEF, leflunomide; MMF, mycophenolate mofetil; MZB, mizoribine; RIT, rituximab; STE, steroids; TAC, tacrolimus; and TAC + MMF, tacrolimus-combined mycophenolate mofetil.

Figure 5 shows testimony that the SUCRA for the 13 therapeutics was 82.8%, 77.2%, 72.4%, 68.3%, 62.9%, 60.0%, 56.5%, 40.4%, 39.4%, 37.3%, 26.0%, 22.7%, and 4.0% for TAC + MMF, TAC, MIZ, ACTH, RIT, CH, CTX, MMF, CsA, LEF, AZA, STE, and CON, respectively. Specific details about results of statistical analysis on the TR are displayed in Supplementary Table S4.

#### 3.5.2 24-h UTP

For the 24-h UTP, 32 RCTs, including 1839 patients, were calculated in the network meta-analysis. The therapeutic schedules involved were as follows: CTX (21 trials, 589 patients), TAC (11, 296), CsA (10, 238), MMF (Polanco et al., 2010; Zotta et al., 2019), RIT (3, 139), LEF (4, 81), STE (2, 99), CH (2, 87), AZA (Haas et al., 1997; Higgins et al., 2012), and CON (7, 231). The network plot is displayed in Figure 2.

The results of network meta-analysis about different immunosuppressants indicated that TAC (standard mean difference (SMD), −2.3, 95% CI (−3.5, −1.1)); CTX (−1.7 (−2.8, −0.59); RIT (−1.8 (−3.5, −0.11); CH (−2.4 (−4.3, −0.49); CsA (−1.7 (−3, −0.49); and AZA (−4.2 (−7.7, −0.68) could significantly superior than the control group, except for LEF (−0.87 (−2.5, 0.77); MMF (−1.2 (−2.7, 0.20)) and STE (−1.0 (−2.8, 0.77) (see Figure 3 and Figure 4 for details).

Unlike the results of NMA on TR, TAC had the highest rate of 24-h UTP (SUCRA of 83.4%). It was followed by RIT (75.7%), AZA (64.7%), CH (57.8%), CTX (54.5%), CsA (50.0%), MMF (41.3%), STE (34.0%), and LEF (33.0%). Meanwhile, CON had the lowest SUCRA value (4.9%) (See Figure 5 and Supplementary Table S4 for details).

#### 3.5.3 Adverse effects

The incidences of adverse events for the 13 therapeutic regimens are shown in Supplementary Table S5. Infections (including respiratory tract infection, urinary tract infection, skin infection, and so on), gastrointestinal symptoms (mainly nausea, vomiting, diarrhea, and epigastric discomfort), bone marrow suppression (mainly leukopenia, anemia, and thrombocytopenia), glucose intolerance, and elevated ALT/AST were the collective adverse events for most immunosuppressants, which was the same as what Zheng et al. (2019) concluded. The incidence of infection had been well documented in an NMA (Liu et al., 2019). Relatively speaking, the four immunosuppressants related to lower frequency of myelosuppression were MIZ (0%), ACTH (0%), TAC (1.14%), and RIT (6.5%), whereas, TAC (22.85%) and CTX (7.11%) were associated with the higher incidence of new-onset diabetes or glucose intolerance. As for infection, MMF (37.5%), RIT (29.58%), and CON (27.16%) are the top three immunosuppressants with the highest percentage. Some less common adverse reactions, such as alopecia, herpes zoster, and malignancy mainly exist in these treatments of CTX, CsA, and CH groups. In addition, it should be noticed that the number of thrombotic attack is four, four, one, and one during the treatment of CTX, CON, STE, and CH, respectively, which had not been reported in other regimens.

### 3.6 Meta-regression and publication bias

Meta-regression was performed to explore the heterogeneity source. As univariate covariates, patients’ age, study duration, and sample size were adjusted for TR and 24-h UTP. The results showed that study duration was associated with the heterogeneity of TR, while there was no significant effect on 24-h UTP. As for patients’ age and sample size, there was no significant effect on either TR or 24-h UTP (see Supplementary File S6 for details). The comparison-adjusted funnel plots were also made, and no significant publication bias was detected (Supplementary File S7).

### 3.7 Sensitivity analyses

For the outcome of TR and 24-h UTP, no significant impact on the overall effect sizes was observed when any single study was omitted according to the sensitivity analysis, indicating the robustness of our results. (See Supplementary File S8).

## 4 Discussion

The incidence of idiopathic membranous nephropathy (IMN) is not low, especially in the middle-aged and elderly. Once the patients are treated improperly or not treated in time, it is likely to lead to the decline of renal function and even progress to end-stage renal disease. In recent 10 years, immunosuppressive agents were applied extensively but controversially in patients with IMN. In this review, we focused on the outcomes most likely to be significant to patients in making treatment decisions, like total remission as well as 24-h UTP and adverse effects. As illustrated in our results based on direct and indirect comparison, compared with CON, TAC + MMF, TAC, RIT, ACTH, CTX, MMF, CsA, and CH demonstrated better therapeutic effect in TR, while TAC, RIT, CTX, CsA, AZA, and CH could significantly superior than the CON group in terms of 24-h UTP. As illustrated in Supplementary File S9, the horizontal axis represents the SUCRA of different treatments on total remission, whereas the longitudinal axis represents the SUCRA of different treatments on 24-h UTP. Combining the SUCRA ranking of TR and 24-h UTP, we can get that significant effect group contains TAC, TAC + MMF, and RIT; the moderate effect group consists of CTX, CH, and MMF; the low effect group includes LEF, CsA, STE, and AZA, and very low effect group includes CON. This aforementioned innovative movement is greatly beneficial for clinicians to choose appropriate immunosuppressants and make wise decisions when facing patients’ different symptoms and needs with idiopathic membranous nephropathy. At the same time, it should be noticed that the four immunosuppressants related to lower frequency of myelosuppression were MIZ, ACTH, TAC, and RIT. Hence, future studies about these immunosuppressants combination, especially the drugs belonging to significant effect group and moderate effect group are pretty necessary to conduct.

The Kidney Disease: Improving Global Outcomes (KDIGO) 2021 guidelines (KDIGO, 2021) recommend rituximab (RIT) therapy, cyclophosphamide CTX) combined steroids (STE) therapy for 6 months, or CNI (CsA or TAC)-based therapy at least 6 months as the initial therapy of IMN with nephrotic syndrome depending on the risk estimate. In our study, TAC and TAC + MMF seem to be significantly superior than CTX or CsA in the increasing total remission rate and decreasing 24-h UTP. In the meantime, we are attracted by an interesting phenomenon that TAC assumes an important role among the treatments in the significant effect group (TAC, TAC + MMF, and RIT). In terms of TR and 24-h UTP, TAC always presented significantly higher probabilities of being in a dominant position, which was the same as what Huang et al. (2021) and Zhu et al. (2017) conducted. Meta-analyses (Zhu et al., 2017) also demonstrate that TAC has superior short-term efficacy and higher safety than CTX within 1 year, but the long-term effects need to be confirmed through further RCTs. The mean treatment duration of our included RCTs was 9.63 months, which reminds us that longer period follow-up studies need to be performed in the further. CsA used to be regarded as a valid alternative to alkylating agents when treating patients with idiopathic membranous nephropathy just in cases of steroid resistance or rapid relapse (Zotta et al., 2019), and the review (Cattran, 2003) expresses the similar views as well, whereas it had no notable advantage over the control group either in total remission or in decreasing 24-h UTP in our review. And then, to make matters worse, the side effects caused by CsA, such as gastrointestinal symptoms, infection, and hypertension, cannot be neglected, which overturns our previous understanding about CsA. RIT, as one of the first choice mentioned in the KDIGO’s Clinical Practice Guideline for IMN in 2021 (69), has been considered as a drug with high remission rate and low relapse rate in treating patients with IMN. In our present study, RIT showed significantly higher probabilities over the control group in TR and 24-h UTP. We can find analogous conclusions from the meta-analysis conducted by You et al. (2021). Meta-analysis (You et al., 2021) also indicated that RIT has obvious advantages in no dependence on steroids and lower rates in relapse and adverse event. However, we should not ignore the economic cost of rituximab at the same time. Compared with other immunosuppressants, the price of rituximab is more expensive, which hinders its popularization in some economically underdeveloped countries to a certain extent. The result of our study also presented that ACTH is beneficial for total remission. Since only one article about ACTH was included, the result of ACTH in the NMA needs to be consolidated by more RCTs. As for AZA and LEF, they had no notable advantages in enhancing TR or decreasing 24-h UTP when compared with the CON group and may produce some less common adverse reactions, such as alopecia and herpes zoster. Thus, the use and promotion of these immunosuppressants in clinical practice requires cautious attitude and careful selection. We also found STE alone had little effect in achieving remission and reducing proteinuria, which explained why regimens of steroids combined with immunosuppressants were often used.

Ren et al. (2017) and Zheng et al. (2019) both published a network meta-analysis of immunosuppressive agents for treating patients with IMN. Our study was more comprehensive than theirs and finally included 51 RCTs with 2,830 participants and 12 immunosuppressants. As far as I am concerned, this study included the largest number of RCTs, largest sample size, and largest variety of therapeutic regimens in estimating the efficacy and safety for IMN in adults. At the same time, this is the first study to obtain comprehensive evaluation of curative effect by integrating the abscissa and ordinate. We have registered this NMA in the website of International Prospective Register of Systematic Reviews in advance and performed a comprehensive literature search. In addition, we performed this NMA complying with the PRISMA guideline. Our findings could provide some evidence on the efficacy and safety of immunosuppressants for patients with IMN.

In addition, several insufficiencies and limitations should be taken into account when interpreting our results. First, the heterogeneity of the included studies was existed, and it is unavoidable, although we have conducted Meta-regression and sensitivity analysis to explore the possible source of heterogeneity, and no significant heterogeneity has been found. Therefore, a cautious interpretation to the results is still necessary. Second, the duration of follow-up varied among the included studies, and some were too short; third, the quality of the included trials was not high. In total 27 (52.9%) studies did not provide enough information on specific randomization methods, which led to selection bias. Fourthly, some studies had a pretty limited sample size, which reduced the level of evidence in our article. Finally, some eligible studies did not provide the details about baseline characteristics, such as blood pressure, blood lipids, and so on, which are regarded as high risk factors for disease progression and decline of renal function, making some difficulties for us to perform the subgroup analysis.

The results of our NMA can offer some evidence for treating IMN in the clinic. Therefore, designing trials more rigorously are extremely needed. More high-quality, large-sample, and multicenter RCTs should be registered prospectively on the corresponding websites to improve the quality of methodology. Moreover, it is advised to provide more details about baseline features as possible, which will provide a good foundation for each NMA. Considering these facts, it is required to confirm the beneficial effects among different immunosuppressive agents for patients with IMN in future research through more well-designed clinical experiments.

## 5 Conclusion

In conclusion, TAC + MMF might be the optimal option in terms of TR, and TAC also performed pretty beneficially for TR. TAC and RIT both show great effectiveness on 24-h UTP compared with other regimens. But when it comes to LEF, AZA, and STE alone, they had no notable advantages in increasing rate of TR or decreasing the level of 24-h UTP when compared with CON and may produce some less common adverse reactions. For all of this, it is required to confirm these findings through more high-quality, large-sample, multicenter RCTs in the future.

## Data Availability

The original contributions presented in the study are included in the article/Supplementary Material; further inquiries can be directed to the corresponding author.

## Abbreviations

IMN, idiopathic membranous nephropathy; NMA, network meta-analysis; RCTs, randomized controlled trials; CTX, cyclophosphamide; MMF, mycophenolate mofetil; TAC + MMF, tacrolimus-combined mycophenolate mofetil; CsA, cyclosporine; TAC, tacrolimus; LEF, leflunomide; CH, chlorambucil; AZA, azathioprine; ACTH, adrenocorticotropic hormone; CON, non-immunosuppressive therapies; STE, steroids; MZB, mizoribine; RIT, rituximab; RRs, risk ratios; SMD, standard mean difference; TR, total remission; 24-h UTP, 24-h urine total protein; SUCRA, surface under the cumulative ranking curve; AEs, adverse events; ALT/AST, alanine aminotransferase or aspartate aminotransferase; and ESRD, end-stage renal disease.

## References

[B1] Branten A. J. Reichert L. J. Koene R. A. Wetzels J. F. (1998). Oral cyclophosphamide versus chlorambucil in the treatment of patients with membranous nephropathy and renal insufficiency. QJM 91 (5), 359–366. 10.1093/qjmed/91.5.359 9709470

[B2] Cameron J. S. Healy M. J. Adu D. (1990). The Medical Research Council trial of short-term high-dose alternate day prednisolone in idiopathic membranous nephropathy with nephrotic syndrome in adults. The MRC Glomerulonephritis Working Party. Q. J. Med. 74 (274), 133–156. 10.1093/oxfordjournals.qjmed.a068422 2189149

[B3] Cattran D. C. Appel G. B. Hebert L. A. Hunsicker L. G. Pohl M. A. Hoy W. E. (2001). Cyclosporine in patients with steroid-resistant membranous nephropathy: A randomized trial. Kidney Int. 59 (4), 1484–1490. 10.1046/j.1523-1755.2001.0590041484.x 11260412

[B4] Cattran D. C. Delmore T. Roscoe J. Cole E. Cardella C. Charron R. (1989). A randomized controlled trial of prednisone in patients with idiopathic membranous nephropathy. N. Engl. J. Med. 320 (4), 210–215. 10.1056/NEJM198901263200403 2643046

[B5] Cattran D. C. (2003). Mycophenolate mofetil and cyclosporine therapy in membranous nephropathy. Semin. Nephrol. 23 (3), 272–277. 10.1016/s0270-9295(03)00051-2 12838495

[B6] Chan T. M. Lin A. W. Tang S. C. Qian J. Q. Lam M. F. Ho Y. W. (2007). Prospective controlled study on mycophenolate mofetil and prednisolone in the treatment of membranous nephropathy with nephrotic syndrome. Nephrol. Carlt. 12 (6), 576–581. 10.1111/j.1440-1797.2007.00822.x 17995584

[B7] Chen M. Li H. Li X. Y. Lu F. M. Ni Z. H. Xu F. F. (2010). Tacrolimus combined with corticosteroids in treatment of nephrotic idiopathic membranous nephropathy: A multicenter randomized controlled trial. Am. J. Med. Sci. 339 (3), 233–238. 10.1097/MAJ.0b013e3181ca3a7d 20220333

[B8] Chen Z. Zhang J. Xia N. He B. (2014). Comparison on the therapeutic effect of cyclosporin A and cyclophosphamide in the treatment of idiopathic membranous nephropathy. China Med. Pharm. 4 (16), 16–18+60.

[B9] Choi J. Y. Kim D. K. Kim Y. W. Yoo T. H. Lee J. P. Chung H. C. (2018). The effect of mycophenolate mofetil versus cyclosporine as combination therapy with low dose corticosteroids in high-risk patients with idiopathic membranous nephropathy: A multicenter randomized trial. J. Korean Med. Sci. 33 (9), e74. 10.3346/jkms.2018.33.e74 29441742 PMC5811664

[B10] Collaborative Study of the Adult Idiopathic Nephrotic Syndrome (1979). A controlled study of short-term prednisone treatment in adults with membranous nephropathy. N. Engl. J. Med. 301 (24), 1301–1306. 10.1056/NEJM197912133012401 388220

[B11] Dahan K. Debiec H. Plaisier E. Cachanado M. Rousseau A. Wakselman L. (2017). Rituximab for severe membranous nephropathy: A 6-month trial with extended follow-up. J. Am. Soc. Nephrol. 28 (1), 348–358. 10.1681/ASN.2016040449 27352623 PMC5198292

[B12] Ding B. (2014). Comparison of the efects of two therapeutic regimen in the treatment of idiopathic membranous nephropathy. Chin. J. Prim. Med. Pharm. 21 (21), 3238–3241.

[B13] Donadio J. V. Jr. Holley K. E. Anderson C. F. Taylor W. F. (1974). Controlled trial of cyclophosphamide in idiopathic membranous nephropathy. Kidney Int. 6 (6), 431–439. 10.1038/ki.1974.129 4613934

[B14] Dong X. Miao J. Chen H. (2017). Comparison of clinical effect on idiopathic membranous nephropathy between cyclosporine A and cyclophosphamide. Pract. J. Cardiac Cereb. Pneumal Vasc. Dis. 25 (S1), 80–81.

[B15] Dussol B. Morange S. Burtey S. Indreies M. Cassuto E. Mourad G. (2008). Mycophenolate mofetil monotherapy in membranous nephropathy: A 1-year randomized controlled trial. Am. J. Kidney Dis. 52 (4), 699–705. 10.1053/j.ajkd.2008.04.013 18585835

[B16] Falk R. J. Hogan S. L. Muller K. E. Jennette J. C. (1992). Treatment of progressive membranous glomerulopathy. A randomized trial comparing cyclophosphamide and corticosteroids with corticosteroids alone. The Glomerular Disease Collaborative Network. Ann. Intern Med. 116 (6), 438–445. 10.7326/0003-4819-116-6-438 1371211

[B17] Fernández-Juárez G. Rojas-Rivera J. Logt A. V. Justino J. Sevillano A. Caravaca-Fontán F. (2021). The STARMEN trial indicates that alternating treatment with corticosteroids and cyclophosphamide is superior to sequential treatment with tacrolimus and rituximab in primary membranous nephropathy. Kidney Int. 99 (4), 986–998. 10.1016/j.kint.2020.10.014 33166580

[B18] Fervenza F. C. Appel G. B. Barbour S. J. Rovin B. H. Lafayette R. A. Aslam N. (2019). Rituximab or cyclosporine in the treatment of membranous nephropathy. N. Engl. J. Med. 381 (1), 36–46. 10.1056/NEJMoa1814427 31269364

[B19] Floege J. Barbour S. J. Cattran D. C. Hogan J. J. Nachman P. H. Tang S. C. W. (2019). Management and treatment of glomerular diseases (part 1): Conclusions from a kidney disease: Improving global outcomes (KDIGO) controversies conference. Kidney Int. 95 (2), 268–280. 10.1016/j.kint.2018.10.018 30665568

[B20] Greco T. Edefonti V. Biondi-Zoccai G. Decarli A. Gasparini M. Zangrillo A. (2015). A multilevel approach to network meta-analysis within a frequentist framework. Contemp. Clin. Trials 42, 51–59. 10.1016/j.cct.2015.03.005 25804722

[B21] Guo Y. Wu X. Liu L. Zhang H. Yang L. Chen W. (2020). Efficacy of leflunomide combined with prednisone for the treatment of PLA2R-associated primary membranous nephropathy. Ren. Fail 42 (1), 122–130. 10.1080/0886022X.2020.1713806 31957527 PMC7006764

[B22] Haas M. Meehan S. M. Karrison T. G. Spargo B. H. (1997). Changing etiologies of unexplained adult nephrotic syndrome: A comparison of renal biopsy findings from 1976-1979 and 1995-1997. Am. J. Kidney Dis. 30 (5), 621–631. 10.1016/s0272-6386(97)90485-6 9370176

[B23] He J. Huang H. Wu J. Li L. Deng X. (2011). Efficacy of leflunomide combined with prednisone acetate in the treatment of idiopathic membranous nephropathy. J. Chin. Pract. Diagnosis Ther. 25 (12), 1218–1219.

[B24] He L. Peng Y. Liu H. Liu Y. Yuan S. Liu F. (2013). Treatment of idiopathic membranous nephropathy with combination of low-dose tacrolimus and corticosteroids. J. Nephrol. 26 (3), 564–571. 10.5301/jn.5000199 22956434

[B25] Higgins J. P. Altman D. G. Gøtzsche P. C. Jüni P. Moher D. Oxman A. D. (2011). The Cochrane Collaboration's tool for assessing risk of bias in randomised trials. BMJ 343, d5928. 10.1136/bmj.d5928 22008217 PMC3196245

[B26] Higgins J. P. Jackson D. Barrett J. K. Lu G. Ades A. E. White I. R. (2012). Consistency and inconsistency in network meta-analysis: Concepts and models for multi-arm studies. Res. Synth. Methods 3 (2), 98–110. 10.1002/jrsm.1044 26062084 PMC4433772

[B27] Hofstra J. M. Fervenza F. C. Wetzels J. F. (2013). Treatment of idiopathic membranous nephropathy. Nat. Rev. Nephrol. 9 (8), 443–458. 10.1038/nrneph.2013.125 23820815

[B28] Howman A. Chapman T. L. Langdon M. M. Ferguson C. Adu D. Feehally J. (2013). Immunosuppression for progressive membranous nephropathy: A UK randomised controlled trial. Lancet 381 (9868), 744–751. 10.1016/S0140-6736(12)61566-9 23312808 PMC3590447

[B29] Huang H. Liang Z. Zheng X. Qing Q. Du X. Tang Z. (2021). Tacrolimus versus cyclophosphamide for patients with idiopathic membranous nephropathy and treated with steroids: A systematic review and meta-analysis of randomized controlled trials. Ren. Fail 43 (1), 840–850. 10.1080/0886022X.2021.1914655 34016023 PMC8158268

[B30] Jha V. Ganguli A. Saha T. K. Kohli H. S. Sud K. Gupta K. L. (2007). A randomized, controlled trial of steroids and cyclophosphamide in adults with nephrotic syndrome caused by idiopathic membranous nephropathy. J. Am. Soc. Nephrol. 18 (6), 1899–1904. 10.1681/ASN.2007020166 17494881

[B31] Jurubita R. Ismail G. Bobeica R. Rusu E. Zilisteanu D. Andronesi A. (2012). Efficacy and safety of triple therapy with MMF, cyclosporine and prednisolone versus cyclosporine and prednisolone in adult patients with idiopathic membranous nephropathy and persistent heavy proteinuria. Nephrol. Dial. Transplant. 27 (Suppl. l_2), ii182–ii96.

[B32] KDIGO (2021). Clinical practice guideline for the management of glomerular diseases. Kidney Int. 100 (4s), S1–s276. 34556256 10.1016/j.kint.2021.05.021

[B33] Kosmadakis G. Filiopoulos V. Smirloglou D. Skarlas P. Georgoulias C. Michail S. (2010). Comparison of immunosuppressive therapeutic regimens in patients with nephrotic syndrome due to idiopathic membranous nephropathy. Ren. Fail 32 (5), 566–571. 10.3109/08860221003728754 20486839

[B34] Li G. Liu T. Bao B. (2011). Comparison on the therapeutic effect of cyclosporin A and cyclophosphamide in the treatment of idiopathic membranous nephropathy. Chin. J. Integr. Traditional West. Nephrol. 12 (06), 522–525.

[B35] Li M. X. Yu Y. W. Zhang Z. Y. Zhao H. D. Xiao F. L. (2015). Administration of low-dose cyclosporine alone for the treatment of elderly patients with membranous nephropathy. Genet. Mol. Res. 14 (1), 2665–2673. 10.4238/2015.March.30.27 25867415

[B36] Li Y. Wang S. Zhao J. Huang Y. (2012). Efficacy and safety of tacrolimus versus cyclosporine in adults wiyh idiopathic membranous nephropathy. Chin. J. Clin. Pharmacol. Ther. 17 (07), 797–801.

[B37] Liang Q. Li H. Xie X. Qu F. Li X. Chen J. (2017). The efficacy and safety of tacrolimus monotherapy in adult-onset nephrotic syndrome caused by idiopathic membranous nephropathy. Ren. Fail 39 (1), 512–518. 10.1080/0886022X.2017.1325371 28562168 PMC6014322

[B38] Liu C. (2014). Investigate on the clinical efficacy of idiopathic membranous treatment with nephropathy mycophenolate mofetil. Chin. J. General Pract. 12 (2), 220–222.

[B39] Liu D. Yang Y. Kuang F. Qing S. Hu B. Yu X. (2019). Risk of infection with different immunosuppressive drugs combined with glucocorticoids for the treatment of idiopathic membranous nephropathy: A pairwise and network meta-analysis. Int. Immunopharmacol. 70, 354–361. 10.1016/j.intimp.2019.03.002 30852290

[B40] Naumovic R. Jovanovic D. Pavlovic S. Stosovic M. Marinkovic J. Basta-Jovanovic G. (2011). Cyclosporine versus azathioprine therapy in high-risk idiopathic membranous nephropathy patients: A 3-year prospective study. Biomed. Pharmacother. 65 (2), 105–110. 10.1016/j.biopha.2010.10.009 21109389

[B41] Nikolopoulou A. Condon M. Turner-Stokes T. Cook H. T. Duncan N. Galliford J. W. (2019). Mycophenolate mofetil and tacrolimus versus tacrolimus alone for the treatment of idiopathic membranous glomerulonephritis: A randomised controlled trial. BMC Nephrol. 20 (1), 352. 10.1186/s12882-019-1539-z 31492152 PMC6731553

[B42] Page M. J. McKenzie J. E. Bossuyt P. M. Boutron I. Hoffmann T. C. Mulrow C. D. (2021). The PRISMA 2020 statement: An updated guideline for reporting systematic reviews. Syst. Rev. 10 (1), 89. 10.1186/s13643-021-01626-4 33781348 PMC8008539

[B43] Peng L. Wei S. Y. Li L. T. He Y. X. Li B. (2016). Comparison of different therapies in high-risk patients with idiopathic membranous nephropathy. J. Formos. Med. Assoc. 115 (1), 11–18. 10.1016/j.jfma.2015.07.021 26315481

[B44] Polanco N. Gutiérrez E. Covarsí A. Ariza F. Carreño A. Vigil A. (2010). Spontaneous remission of nephrotic syndrome in idiopathic membranous nephropathy. J. Am. Soc. Nephrol. 21 (4), 697–704. 10.1681/ASN.2009080861 20110379 PMC2844306

[B45] Ponticelli C. Altieri P. Scolari F. Passerini P. Roccatello D. Cesana B. (1998). A randomized study comparing methylprednisolone plus chlorambucil versus methylprednisolone plus cyclophosphamide in idiopathic membranous nephropathy. J. Am. Soc. Nephrol. 9 (3), 444–450. 10.1681/ASN.V93444 9513907

[B46] Ponticelli C. Passerini P. Salvadori M. Manno C. Viola B. F. Pasquali S. (2006). A randomized pilot trial comparing methylprednisolone plus a cytotoxic agent versus synthetic adrenocorticotropic hormone in idiopathic membranous nephropathy. Am. J. Kidney Dis. 47 (2), 233–240. 10.1053/j.ajkd.2005.10.016 16431252

[B47] Ponticelli C. Zucchelli P. Passerini P. Cagnoli L. Cesana B. Pozzi C. (1989). A randomized trial of methylprednisolone and chlorambucil in idiopathic membranous nephropathy. N. Engl. J. Med. 320 (1), 8–13. 10.1056/NEJM198901053200102 2642605

[B48] Ponticelli C. Zucchelli P. Passerini P. Cesana B. Locatelli F. Pasquali S. (1995). A 10-year follow-up of a randomized study with methylprednisolone and chlorambucil in membranous nephropathy. Kidney Int. 48 (5), 1600–1604. 10.1038/ki.1995.453 8544420

[B49] Ponticelli C. Zucchelli P. Passerini P. Cesana B. (1992). Methylprednisolone plus chlorambucil as compared with methylprednisolone alone for the treatment of idiopathic membranous nephropathy. The Italian Idiopathic Membranous Nephropathy Treatment Study Group. N. Engl. J. Med. 327 (9), 599–603. 10.1056/NEJM199208273270904 1640953

[B50] Praga M. Barrio V. Juárez G. F. Luño J. (2007). Tacrolimus monotherapy in membranous nephropathy: A randomized controlled trial. Kidney Int. 71 (9), 924–930. 10.1038/sj.ki.5002215 17377504

[B51] Ramachandran R. Hn H. K. Kumar V. Nada R. Yadav A. K. Goyal A. (2016). Tacrolimus combined with corticosteroids versus Modified Ponticelli regimen in treatment of idiopathic membranous nephropathy: Randomized control trial. Nephrol. Carlt. 21 (2), 139–146. 10.1111/nep.12569 26205759

[B52] Ramachandran R. Yadav A. K. Kumar V. Siva Tez Pinnamaneni V. Nada R. Ghosh R. (2017). Two-year follow-up study of membranous nephropathy treated with tacrolimus and corticosteroids versus cyclical corticosteroids and cyclophosphamide. Kidney Int. Rep. 2 (4), 610–616. 10.1016/j.ekir.2017.02.004 29142979 PMC5678834

[B53] Reichert L. J. Huysmans F. T. Assmann K. Koene R. A. Wetzels J. F. (1994). Preserving renal function in patients with membranous nephropathy: Daily oral chlorambucil compared with intermittent monthly pulses of cyclophosphamide. Ann. Intern Med. 121 (5), 328–333. 10.7326/0003-4819-121-5-199409010-00003 8042821

[B54] Ren S. Wang Y. Xian L. Toyama T. Jardine M. Li G. (2017). Comparative effectiveness and tolerance of immunosuppressive treatments for idiopathic membranous nephropathy: A network meta-analysis. PloS one 12 (9), e0184398. 10.1371/journal.pone.0184398 28898290 PMC5595305

[B55] Rosenzwajg M. Languille E. Debiec H. Hygino J. Dahan K. Simon T. (2017). B- and T-cell subpopulations in patients with severe idiopathic membranous nephropathy may predict an early response to rituximab. Kidney Int. 92 (1), 227–237. 10.1016/j.kint.2017.01.012 28318628

[B56] Salanti G. Ades A. E. Ioannidis J. P. (2011). Graphical methods and numerical summaries for presenting results from multiple-treatment meta-analysis: An overview and tutorial. J. Clin. Epidemiol. 64 (2), 163–171. 10.1016/j.jclinepi.2010.03.016 20688472

[B57] Salanti G. (2012). Indirect and mixed-treatment comparison, network, or multiple-treatments meta-analysis: Many names, many benefits, many concerns for the next generation evidence synthesis tool. Res. Synth. Methods 3 (2), 80–97. 10.1002/jrsm.1037 26062083

[B58] Scolari F. Delbarba E. Santoro D. Gesualdo L. Pani A. Dallera N. (2021). Rituximab or cyclophosphamide in the treatment of membranous nephropathy: The RI-CYCLO randomized trial. Jasn. 32 (4), 972–982. 10.1681/asn.2020071091 PMC8017548 33649098

[B59] Senthil Nayagam L. Ganguli A. Rathi M. Kohli H. S. Gupta K. L. Joshi K. (2008). Mycophenolate mofetil or standard therapy for membranous nephropathy and focal segmental glomerulosclerosis: A pilot study. Nephrol. Dial. Transpl. 23 (6), 1926–1930. 10.1093/ndt/gfm538 17989103

[B60] Shibasaki T. Koyama A. Hishida A. Muso E. Osawa G. Yamabe H. (2004). A randomized open-label comparative study of conventional therapy versus mizoribine onlay therapy in patients with steroid-resistant nephrotic syndrome (postmarketing survey). Clin. Exp. Nephrol. 8 (2), 117–126. 10.1007/s10157-004-0276-0 15235928

[B61] Sun G. Xu Z. Luo P. Miao L. (2008). Clinical effect of tacrolimus in the treatment of idiopathic membranous nephropathy. Chin. J. Gerontology 28 (5), 469–471.

[B62] Tonin F. S. Rotta I. Mendes A. M. Pontarolo R. (2017). Network meta-analysis: A technique to gather evidence from direct and indirect comparisons. Pharm. Pract. (Granada) 15 (1), 943. 10.18549/PharmPract.2017.01.943 28503228 PMC5386629

[B63] van de Logt A. E. Hofstra J. M. Wetzels J. F. (2016). Pharmacological treatment of primary membranous nephropathy in 2016. Expert Rev. Clin. Pharmacol. 9 (11), 1463–1478. 10.1080/17512433.2016.1225497 27535699

[B64] Veroniki A. A. Vasiliadis H. S. Higgins J. P. Salanti G. (2013). Evaluation of inconsistency in networks of interventions. Int. J. Epidemiol. 42 (1), 332–345. 10.1093/ije/dys222 23508418 PMC5411010

[B65] Wu Q. Gong Z. (2011). Clinical observation on 20 cases of membranous nephropathy treated with medium and small dose cyclosporine. China Pharm. 14 (1), 115–117.

[B66] Wu Y. Zuo K. Wang B. Li S. Liu Z. (2012). Combination therapy of prednisone and cyclophosphamide for patients with idiopathic membranous nephropathy:a prospective randomized controlled trial. Chin. J. Nephrol. Dialysis Transplant. 21 (2), 109–114.

[B67] Xu J. Zhang W. Xu Y. Shen P. Ren H. Wang W. (2013). Tacrolimus combined with corticosteroids in idiopathic membranous nephropathy: A randomized, prospective, controlled trial. Contrib. Nephrol. 181, 152–162. 10.1159/000348475 23689577

[B68] Xue X. (2013). Clinical observation of tacrolimus in the treatment of 30 cases of membranous nephropathy [master]. Zhengzhou, China: Zhengzhou University.

[B69] Yao X. Chen H. Tang Z. Hu W. Yin G. Liu Z. (1997). Cyclosporin A in idiopathic membranous nephropathy: A prospective clinical trial. Chin. J. Nephrol. Dialysis Transplant. 102 (2), 22–27.

[B70] You L. Ye P. Xiao G. Liang J. Kong Y. (2021). Rituximab for the treatment of idiopathic membranous nephropathy with nephrotic syndrome: A systematic review and meta-analysis. Turk J. Med. Sci. 51 (6), 2870–2880. 10.3906/sag-2104-177 34391323 PMC10734821

[B71] Yu-Kang T. (2016). Node-splitting generalized linear mixed models for evaluation of inconsistency in network meta-analysis. Value Health 19 (8), 957–963. 10.1016/j.jval.2016.07.005 27987646

[B72] Zheng Q. Yang H. Liu W. Sun W. Zhao Q. Zhang X. (2019). Comparative efficacy of 13 immunosuppressive agents for idiopathic membranous nephropathy in adults with nephrotic syndrome: A systematic review and network meta-analysis. BMJ Open 9 (9), e030919. 10.1136/bmjopen-2019-030919 PMC6738938 31511292

[B73] Zhu L. B. Liu L. L. Yao L. Wang L. N. (2017). Efficacy and safety of tacrolimus versus cyclophosphamide for primary membranous nephropathy: A meta-analysis. Drugs 77 (2), 187–199. 10.1007/s40265-016-0683-z 28084563

[B74] Zotta F. Di Stasio E. Manzione A. Pirozzi N. Stoppacciaro A. Menè P. (2019). Steroid and cyclosporine therapy in idiopathic membranous nephropathy: Monocentric experience and literature review. G. Ital. Nefrol. 36 (3). 31251003

