# Supplementary material for: Efficacy and safety of 12 immunosuppressive agents for idiopathic membranous nephropathy in adults: A pairwise and network meta-analysis
Source: Front Pharmacol. 2022 Jul 25;13:917532. doi: 10.3389/fphar.2022.917532 (PMC9358043; doi:10.3389/fphar.2022.917532)
Supplement: Supplementary file 1 [file Table4.doc]

| **Table 2 SUCRA value of total remission and 24 hours UTP** | | | | | | |
| --- | --- | --- | --- | --- | --- | --- |
|  | **Total remission** | | | **24 hours UTP** | | |
| **Treatments** | **SUCRA** | **PrBest** | **Mean rank** | **SUCRA** | **PrBest** | **Mean rank** |
| **CTX** | 56.5 | 0.0 | 6.2 | 54.5 | 0.1 | 5.1 |
| **CsA** | 39.4 | 0.1 | 8.3 | 50.6 | 0.6 | 5.4 |
| **TAC** | 77.2 | 2.4 | 3.7 | 83.4 | 23.9 | 2.5 |
| **RIT** | 62.9 | 2.4 | 5.5 | 75.7 | 23.2 | 3.2 |
| **STE** | 22.7 | 0.1 | 10.3 | 34.0 | 2.9 | 6.9 |
| **CON** | 4.0 | 0.0 | 12.5 | 4.9 | 0.0 | 9.6 |
| **CH** | 60.0 | 2.1 | 5.8 | 57.8 | 15.2 | 4.8 |
| **MMF** | 40.4 | 0.2 | 8.1 | 41.3 | 1.1 | 6.3 |
| **LEF** | 37.3 | 0.3 | 8.5 | 33.0 | 0.8 | 7.0 |
| **AZA** | 26.0 | 1.7 | 9.9 | 64.7 | 32.3 | 4.2 |
| **MIZ** | 72.4 | 52.1 | 4.3 | NR | NR | NR |
| **ACTH** | 68.3 | 12.3 | 4.8 | NR | NR | NR |
| **TAC+MMF** | 82.8 | 26.3 | 3.1 | NR | NR | NR |

Notes: CON, non-immunosuppressive therapies (the control group); CsA, cyclosporine; CTX, cyclophosphamide; ACTH, adrenocorticotropic hormone; AZA, azathioprine; CH, chlorambucil; LEF, leflunomide; MMF, mycophenolate mofetil; MZB, mizoribine; RIT, rituximab; STE, steroids; TAC, tacrolimus; TAC+MMF, tacrolimus combined mycophenolate mofetil; NR, not reported.
