# Supplementary material for: Efficacy and safety of 12 immunosuppressive agents for idiopathic membranous nephropathy in adults: A pairwise and network meta-analysis
Source: Front Pharmacol. 2022 Jul 25;13:917532. doi: 10.3389/fphar.2022.917532 (PMC9358043; doi:10.3389/fphar.2022.917532)

***Supplementary File 4: Evaluation of heterogeneity analysis.***

1. Total Remission


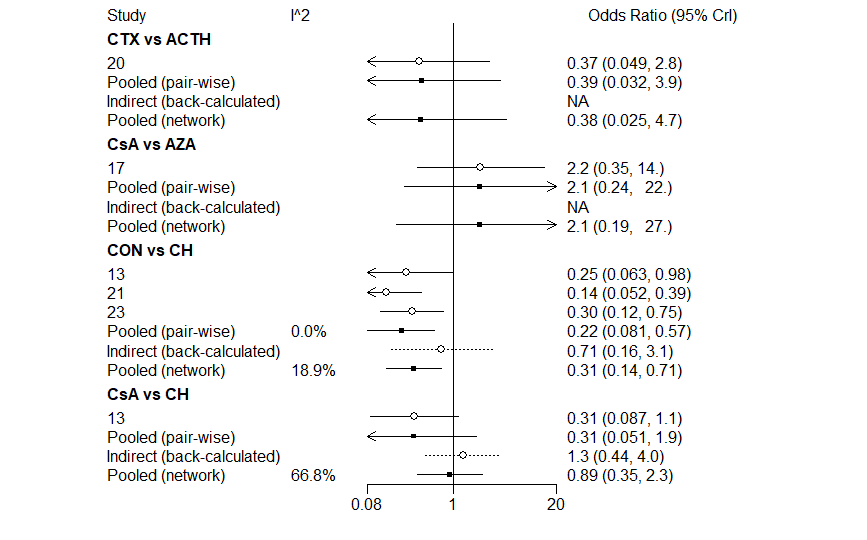


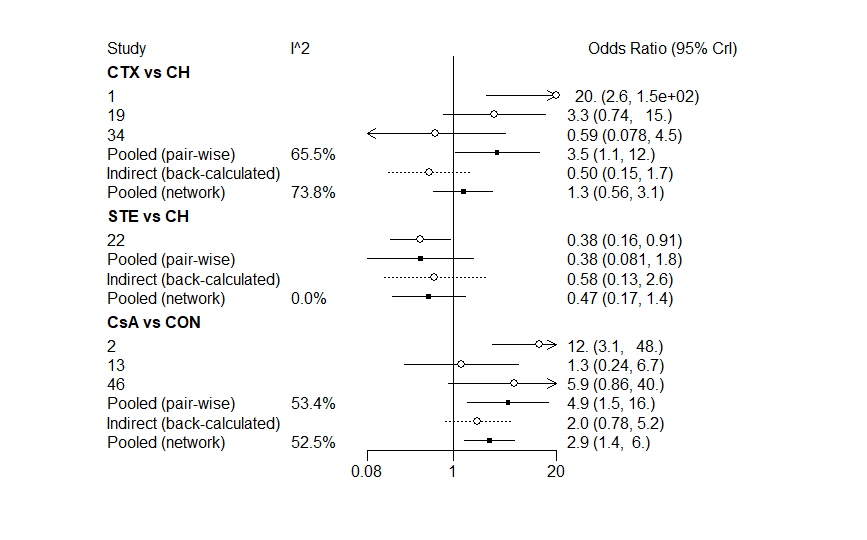


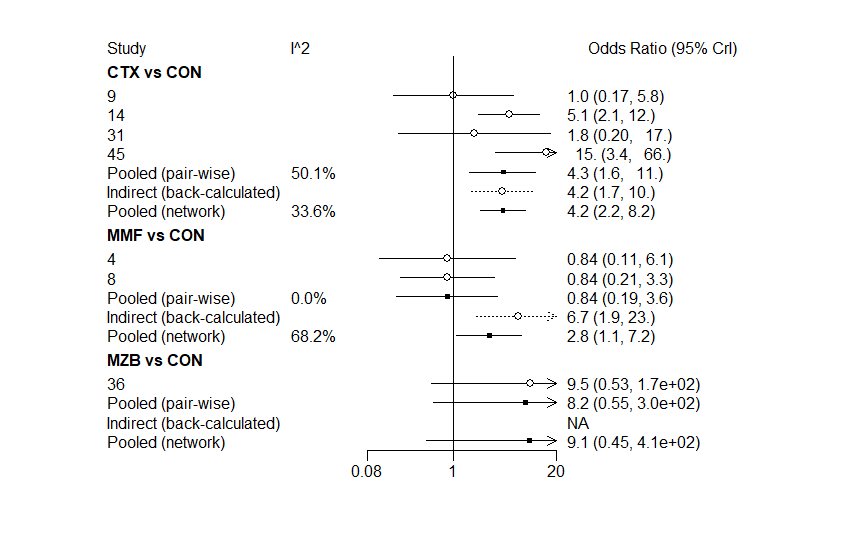


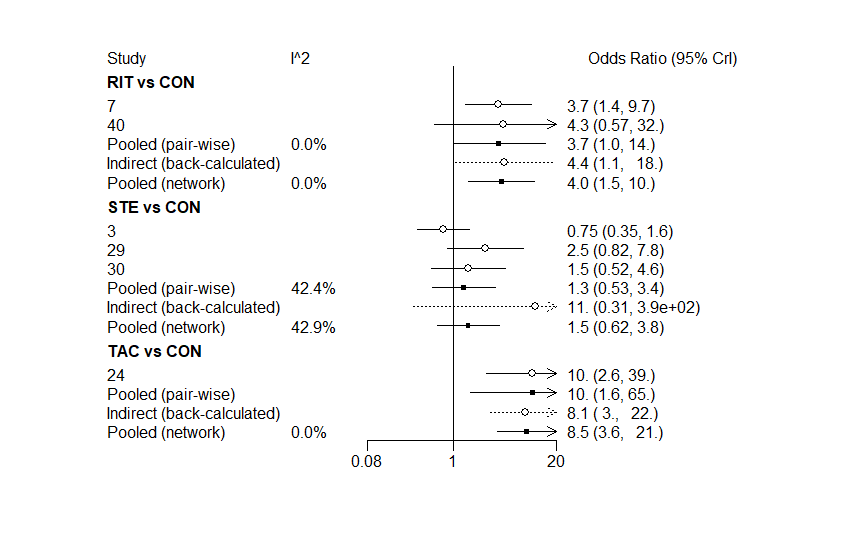


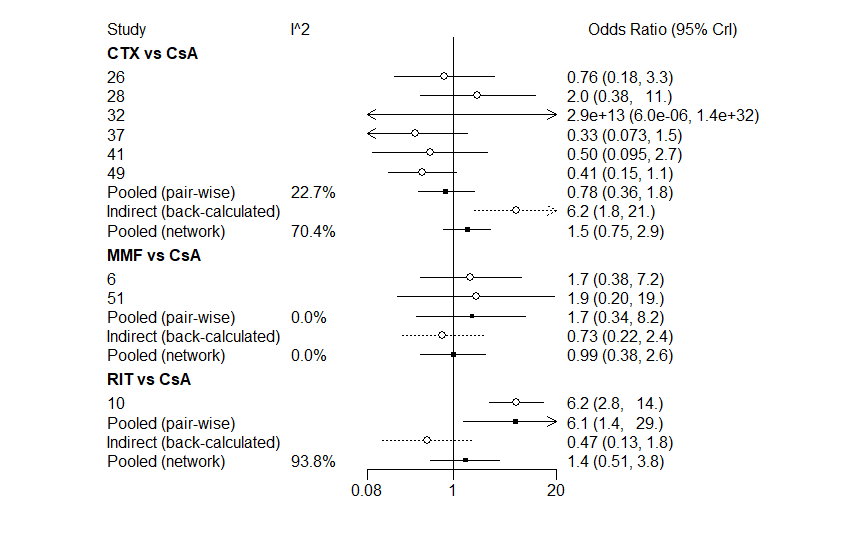


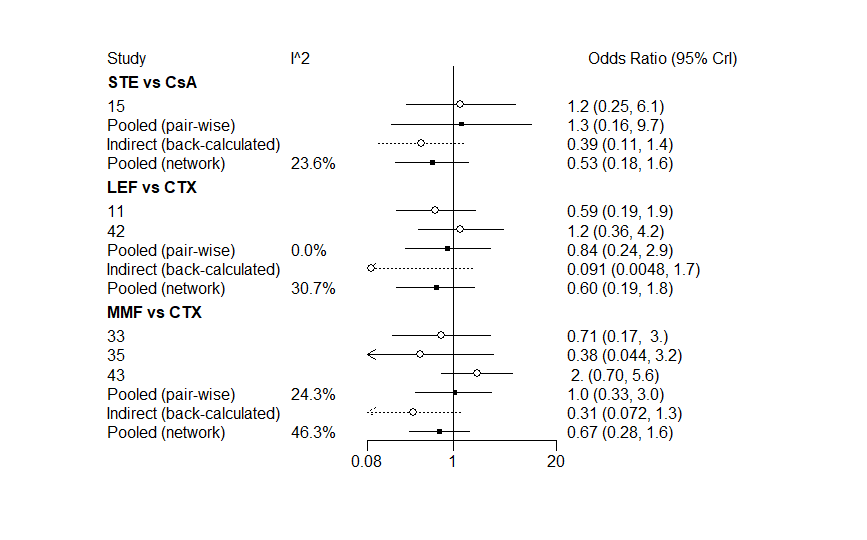


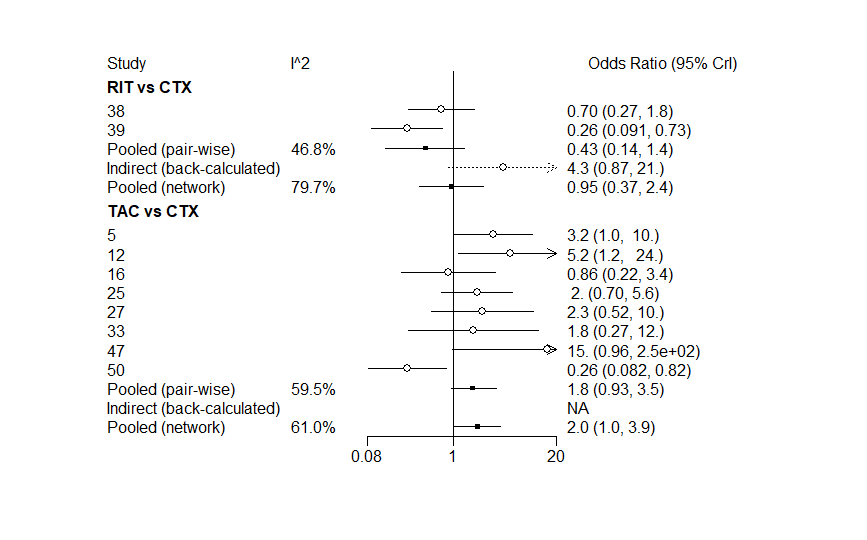


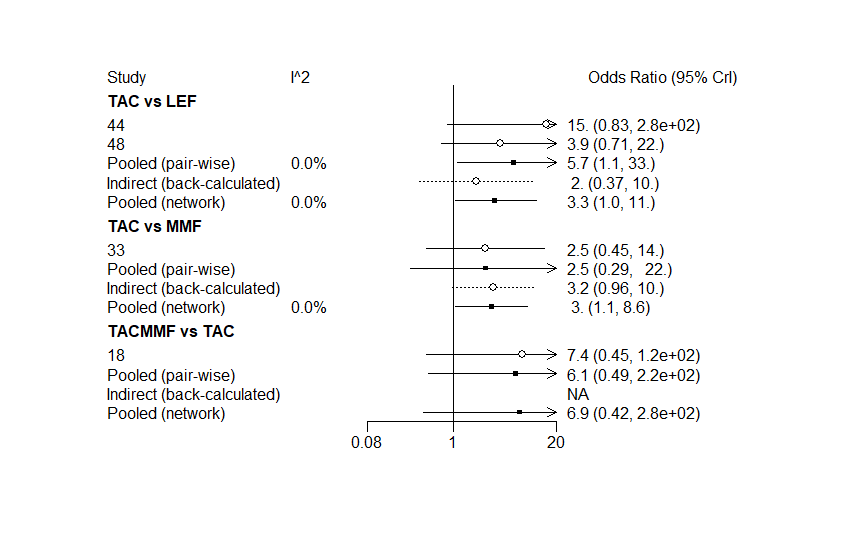


2. 24 Hours Urine Total Protein


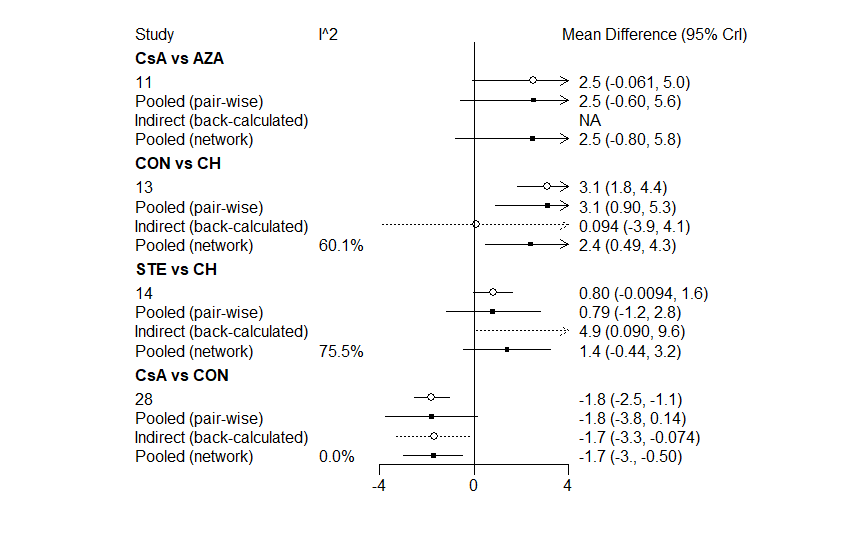


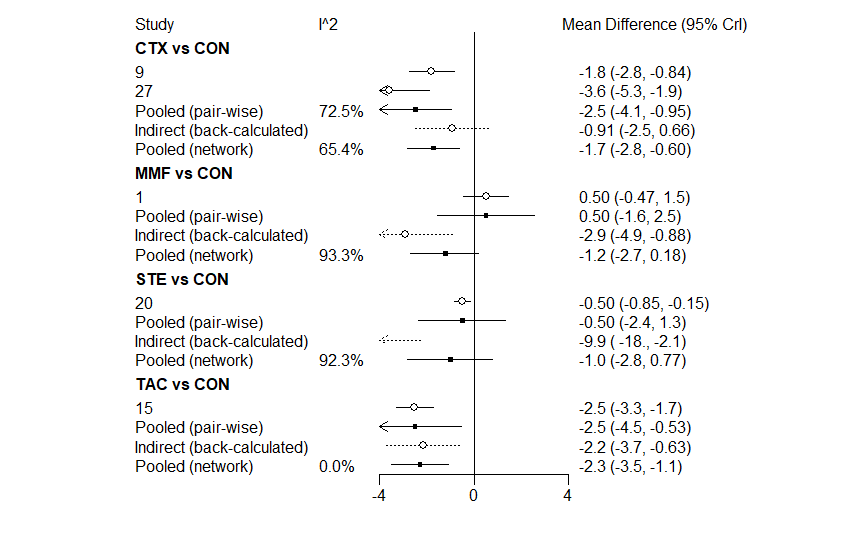


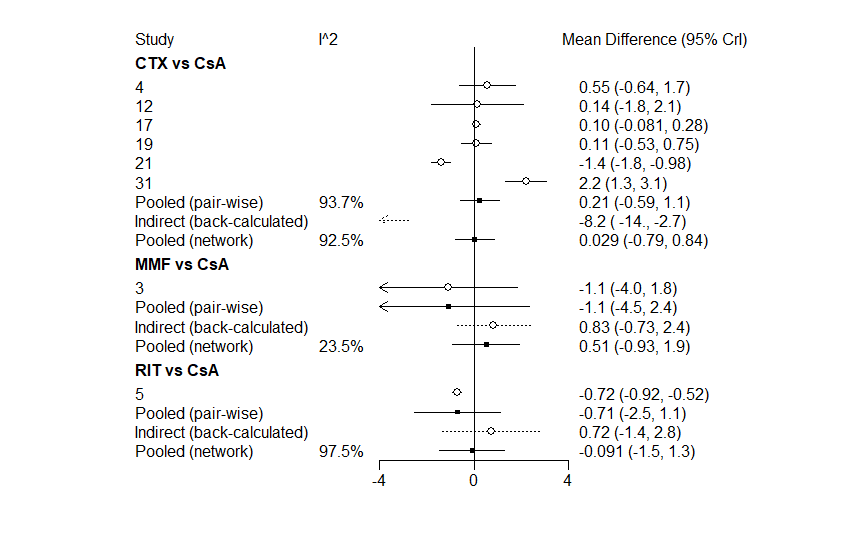


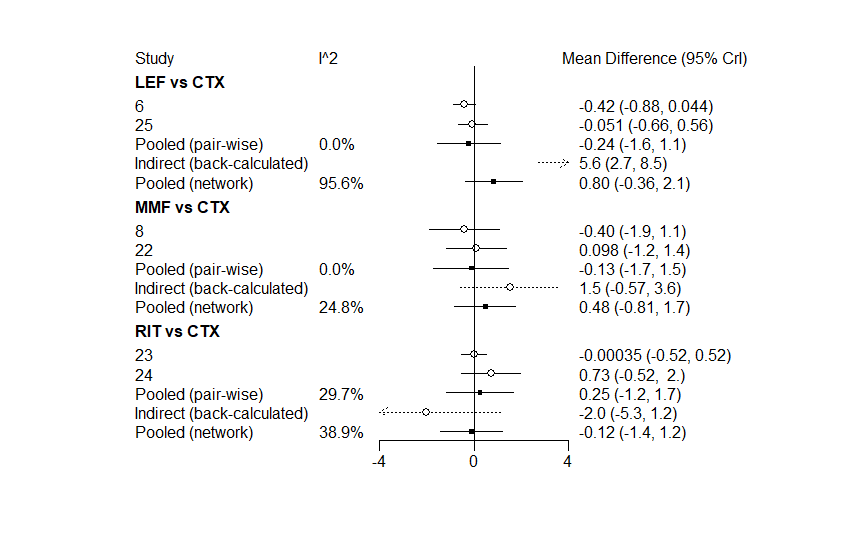


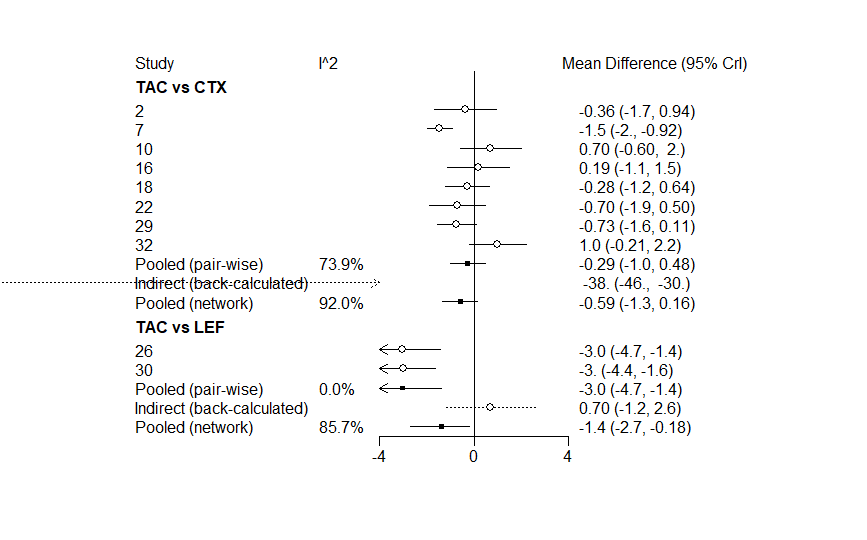


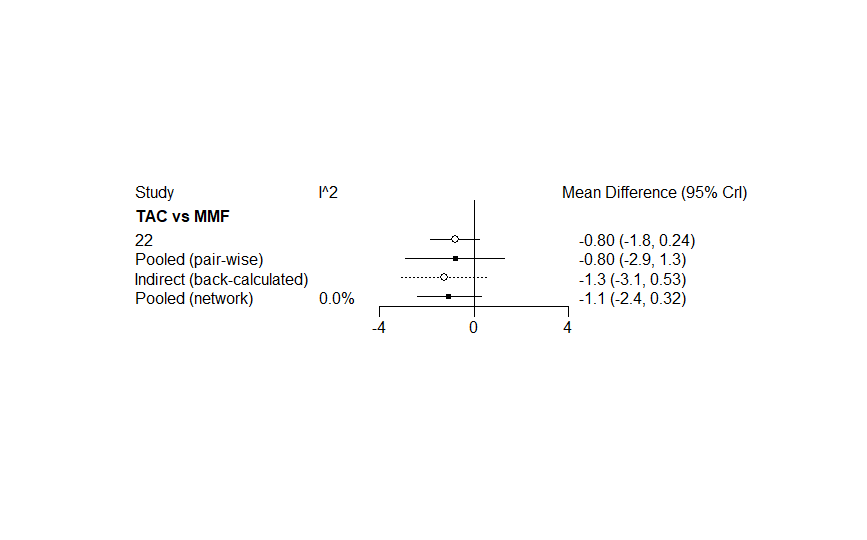

Supplement: Supplementary file 2 [file DataSheet4.docx]
