# Supplementary material for: Efficacy and safety of 12 immunosuppressive agents for idiopathic membranous nephropathy in adults: A pairwise and network meta-analysis
Source: Front Pharmacol. 2022 Jul 25;13:917532. doi: 10.3389/fphar.2022.917532 (PMC9358043; doi:10.3389/fphar.2022.917532)
Supplement: Supplementary file 3 [file DataSheet7.docx]

***Supplementary File 7: Net-funnel plot of Publication bias***

1. **Total remission**

**
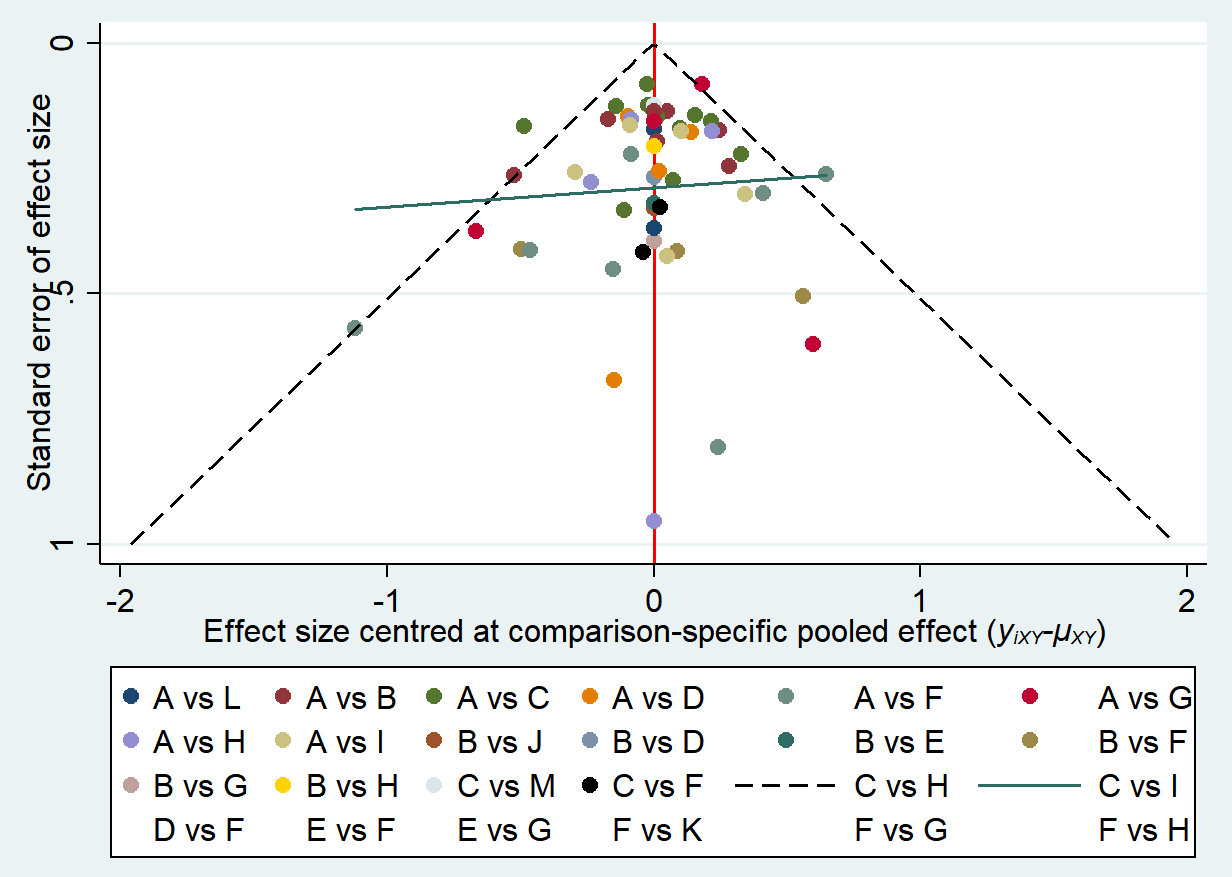
eFig.3** Funnel plots of total remission. A, cyclophosphamide; B, cyclosporine; C, tacrolimus; D, rituximab; E, steroids; F, non-immunosuppressive therapies (the control group); G, chlorambucil; H, mycophenolate mofetil; I, leflunomide; J, azathioprine; K, mizoribine; L, adrenocorticotropic hormone; M, tacrolimus combined mycophenolate mofetil.

1. **24 hours urine total protein**

**
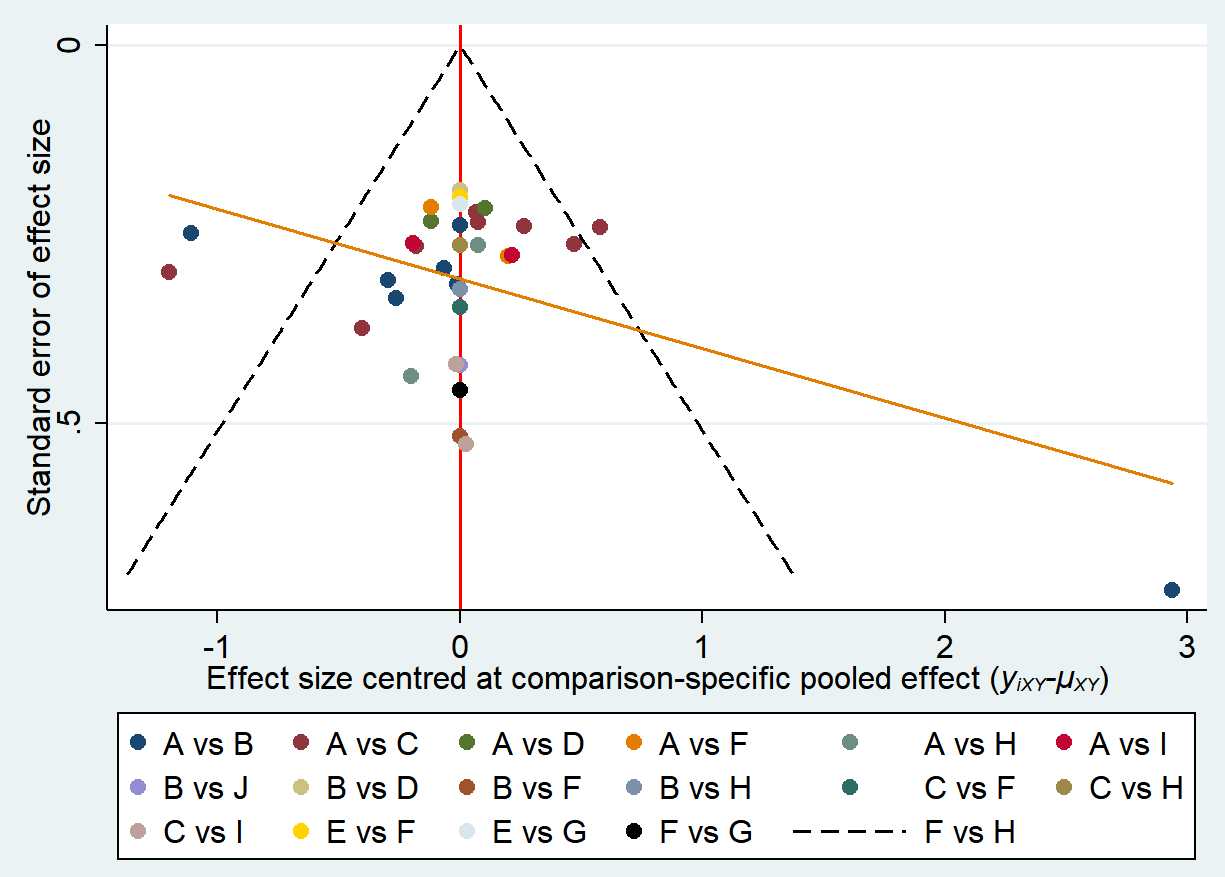
eFig.4** Funnel plots of 24 hours urine total protein. A, cyclophosphamide; B, cyclosporine; C, tacrolimus; D, rituximab; E, steroids; F, non-immunosuppressive therapies (the control group); G, chlorambucil; H, mycophenolate mofetil; I, leflunomide; J, azathioprine; K, mizoribine; L, adrenocorticotropic hormone; M, tacrolimus combined mycophenolate mofetil.
