# Supplementary material for: Efficacy and safety of 12 immunosuppressive agents for idiopathic membranous nephropathy in adults: A pairwise and network meta-analysis
Source: Front Pharmacol. 2022 Jul 25;13:917532. doi: 10.3389/fphar.2022.917532 (PMC9358043; doi:10.3389/fphar.2022.917532)
Supplement: Supplementary file 4 [file Table1.docx]

e Table 1 The selection criteria with a “PICOS” structure for the enrolled studies:

| Items | Specific Criteria |
| --- | --- |
| Patients | Patients with biopsy proven IMN and nephrotic range proteinuria (urinary protein excretion >3.5g/24h) and all included participants had a study-duration of at least 6 months. |
| Interventions/Comparisons | Interventions/Comparisons included CTX, CsA, TAC, RIT, STE, CON, CH, MMF, LEF, AZA, MIZ, ACTH, TAC+MMF |
| Outcomes | Outcomes were Total remission (TR), 24 hours urine total protein (24h UTP) and adverse effects. |
| Study designs | Studies were randomized controlled trials (RCTs) |

ACTH, adrenocorticotropic hormone; AZA, azathioprine; CH, chlorambucil; CON, non-immunosuppressive therapies (the control group); CsA, cyclosporine; CTX, cyclophosphamide; LEF, leflunomide; MMF, mycophenolate mofetil; MZB, mizoribine; RIT, rituximab; STE, steroids; TAC, tacrolimus; TAC+MMF, tacrolimus combined mycophenolate mofetil.
