# Supplementary material for: Efficacy and safety of 12 immunosuppressive agents for idiopathic membranous nephropathy in adults: A pairwise and network meta-analysis
Source: Front Pharmacol. 2022 Jul 25;13:917532. doi: 10.3389/fphar.2022.917532 (PMC9358043; doi:10.3389/fphar.2022.917532)

***Supplementary File 3: Evaluation of inconsistency for outcomes***

1. **Total Remission**


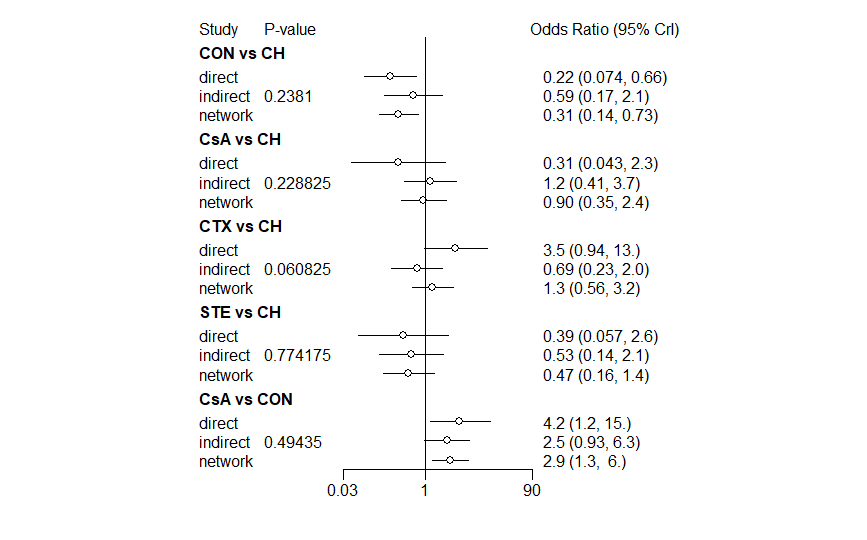


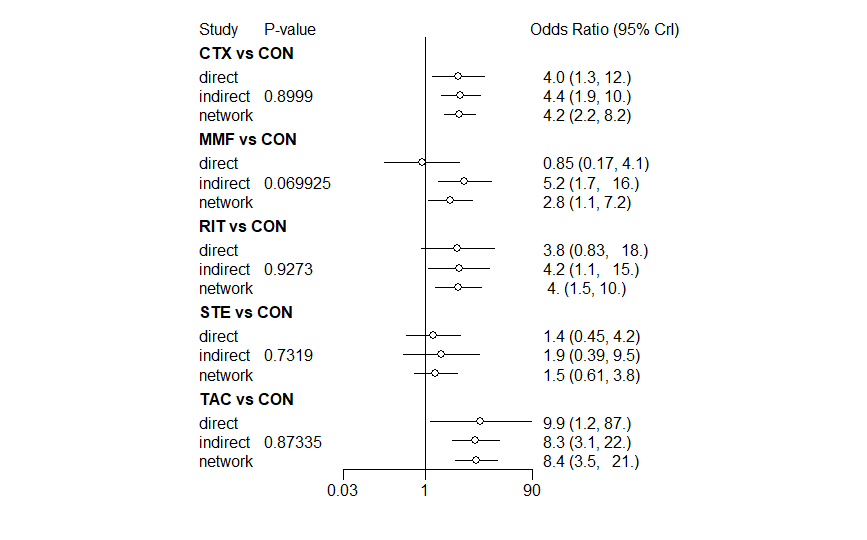


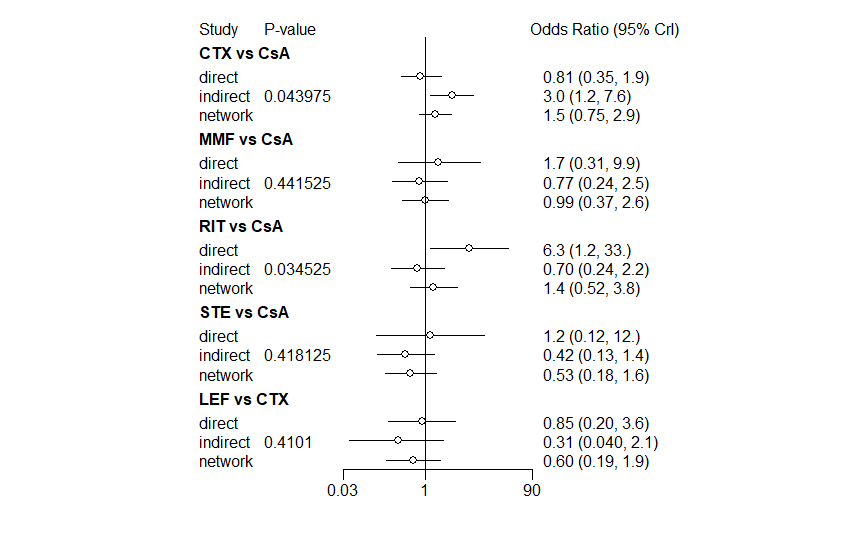


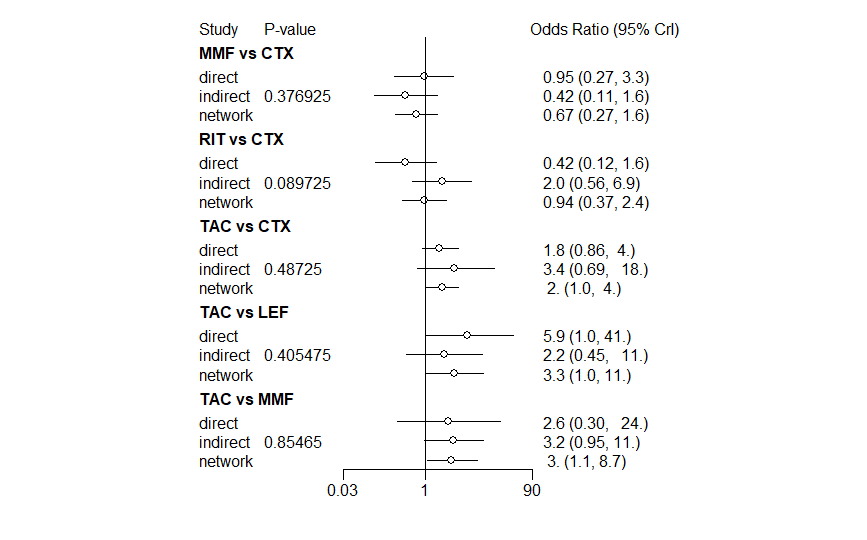


1. **24 Hours Urine Total Protein**


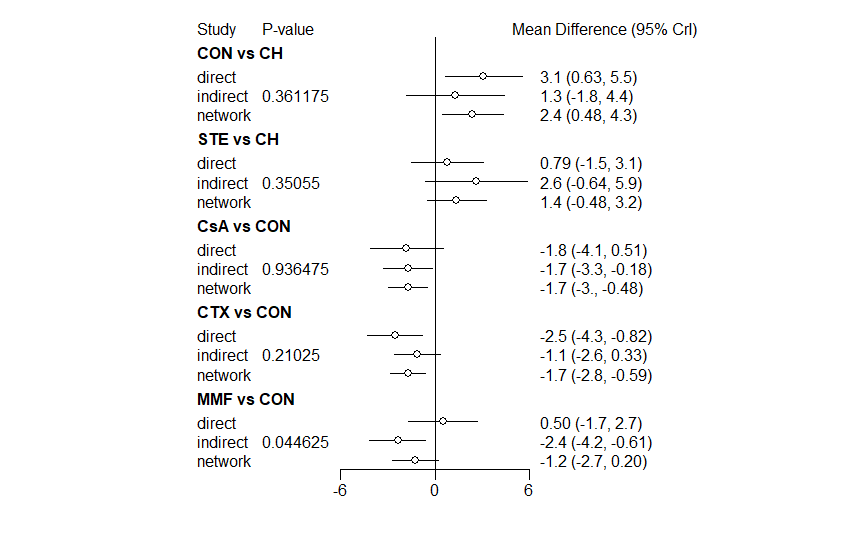


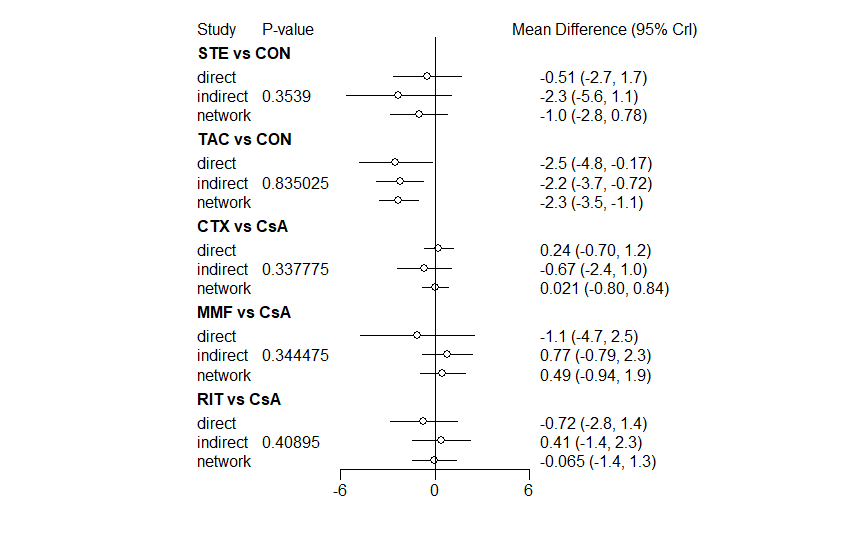


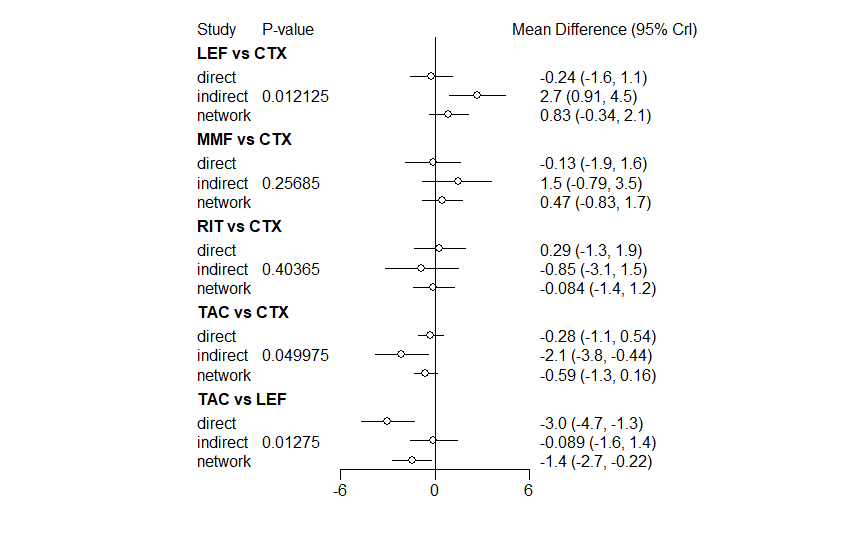


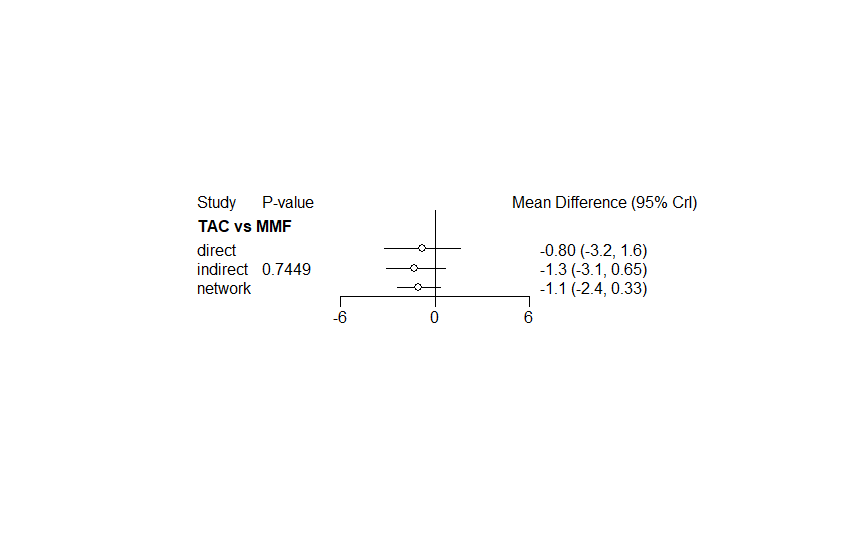

Supplement: Supplementary file 5 [file DataSheet3.docx]
