# Supplementary material for: Efficacy and safety of 12 immunosuppressive agents for idiopathic membranous nephropathy in adults: A pairwise and network meta-analysis
Source: Front Pharmacol. 2022 Jul 25;13:917532. doi: 10.3389/fphar.2022.917532 (PMC9358043; doi:10.3389/fphar.2022.917532)
Supplement: Supplementary file 6 [file DataSheet5.docx]

***Supplementary File 5 : Results from pairwise meta-analyses***

eTable 1 Pairwise meta-analysis results of total remission.

| **Contrast** | **No. of study** | **RR** | **95%CI** | **Q** | **I^2^** | **tau^2^** | **P** |
| --- | --- | --- | --- | --- | --- | --- | --- |
| CTX vs CH | 3 | 3.08 | (0.597, 15.888) | 0.062 | 64.1% | 1.3443 | 0.179 |
| CTX vs TAC | 8 | 0.893 | (0.773, 1.032) | 15.50 | 54.8% | 0.0226 | 0.124 |
| CTX vs CON | 4 | 3.731 | (1.349, 10.318) | 0.104 | 51.3% | 0.5396 | 0.011 |
| CTX vs MMF | 3 | 0.944 | (0.408, 2.187) | 0.286 | 20.2% | 0.1166 | 0.893 |
| CTX vs LEF | 2 | 1.052 | (0.831, 1.331) | 0.65 | 0.0% | 0.0000 | 0.676 |
| CTX vs ACTH | 1 | 0.429 | (0.066, 2.765) | 0 | - | - | 0.373 |
| CTX vs CsA | 6 | 0.947 | (0.777, 1.155) | 8.910 | 43.9% | 0.0261 | 0.592 |
| CTX vs RIT | 2 | 2.257 | (0.883, 5.770) | 0.176 | 45.4% | 0.2084 | 0.089 |
| CsA vs CON | 3 | 3.919 | (0.971, 15.826) | 0.054 | 65.6% | 0.9898 | 0.055 |
| CsA vs MMF | 2 | 0.869 | (0.624, 1.211) | 0 | 0.0% | 0.0000 | 0.407 |
| CsA vs RIT | 1 | 0.167 | (0.067, 0.365) | 0 | - | - | 0.000 |
| CsA vs CH | 1 | 0.328 | (0.112, 0.961) | 0 | - | - | 0.042 |
| CsA vs AZA | 1 | 0.333 | (0.026, 4.319) | 0 | - | - | 0.401 |
| CsA vs STE | 1 | 0.833 | (0.179, 3.884) | 0 | - | - | 0.816 |
| TAC vs CON | 1 | 8.972 | (2.428, 33.158) | 0 | - | - | 0.001 |
| TAC vs TAC+MMF | 1 | 0.211 | (0.021, 2.079) | 0 | - | - | 0.182 |
| TAC vs MMF | 1 | 2.381 | (0.628, 9.030) | 0 | - | - | 0.202 |
| TAC vs LEF | 2 | 4.698 | (1.224, 18.038) | 0.523 | 0.0% | 0.0000 | 0.024 |
| RIT vs CON | 2 | 3.540 | (1.519, 8.249) | 0.989 | 0.0% | 0.0000 | 0.003 |
| STE vs CON | 3 | 1.274 | (0.633,2.565) | 0.196 | 38.7% | 0.1495 | 0.497 |
| STE vs CH | 1 | 0.391 | (0.166, 0.917) | 0 | - | - | 0.031 |
| CON vs MMF | 2 | 1.180 | (0.398, 3.499) | 0.967 | 0.0% | 0.0000 | 0.765 |
| CON vs CH | 3 | 0.231 | (0.130, 0.410) | 0.557 | 0.0% | 0.0000 | 0.000 |
| CON vs MIZ | 1 | 0.167 | (0.014, 1.938) | 0 | - | - | 0.152 |

Note: ACTH, adrenocorticotropic hormone; AZA, azathioprine; CH, chlorambucil; CON, non-immunosuppressive

therapies (the control group); CsA, cyclosporine; CTX, cyclophosphamide; LEF, leflunomide; MMF, mycophenolate

mofetil; MZB, mizoribine; RIT, rituximab; STE, steroids; TAC, tacrolimus; TAC+MMF, tacrolimus combined

mycophenolate mofetil.

eTable 1 Pairwise meta-analysis results of 24 hours urine total protein.

| **Contrast** | **No. of study** | **SMD** | **95%CI** | **Q** | **I^2^** | **tau^2^** | **P** |
| --- | --- | --- | --- | --- | --- | --- | --- |
| CTX vs CsA | 6 | 0.239 | (-0.640, 1.118) | 70.97 | 93.0% | 0.9896 | 0.594 |
| CTX vs TAC | 8 | 0.311 | (-0.305, 0.927) | 22.34 | 68.7% | 0.5125 | 0.323 |
| CTX vs RIT | 2 | -0.120 | (-0.431, 0.190) | 0.50 | 0.0% | 0.0000 | 0.448 |
| CTX vs CON | 2 | -0.885 | (-1.219, -0.551) | 0.84 | 0.0% | 0.0000 | 0.000 |
| CTX vs MMF | 2 | 0.023 | (-0.422, 0.467) | 0.30 | 0.0% | 0.0000 | 0.920 |
| CTX vs LEF | 2 | 0.286 | (-0.083, 0.656) | 0.89 | 0.0% | 0.0000 | 0.129 |
| CsA vs RIT | 1 | 1.252 | (0.875, 1.628) | 0.00 | - | - | 0.000 |
| CsA vs CON | 1 | -1.896 | (-2.900, -0.892) | 0.00 | - | - | 0.000 |
| CsA vs MMF | 1 | 0.245 | (-0.387,0.877) | 0.00 | - | - | 0.447 |
| CsA vs AZA | 1 | 0.882 | (0.015, 1.748) | 0.00 | - | - | 0.000 |
| TAC vs CON | 1 | -1.822 | (-2.499, -1.144) | 0.00 | - | - | 0.000 |
| TAC vs MMF | 1 | -0.457 | (-0.979, 0.065) | 0.00 | - | - | 0.086 |
| TAC vs LEF | 2 | -1.587 | (-2.230, -0.945) | 0.01 | 0.0% | 0.0000 | 0.000 |
| STE vs CON | 1 | -0.552 | (-0.945, -0.158) | 0.00 | - | - | 0.006 |
| STE vs CH | 1 | 0.402 | (-0.011, 0.815) | 0.00 | - | - | 0.056 |
| CON vs CH | 1 | 1.064 | (0.598, 1.530) | 0.00 | - | - | 0.000 |
| CON vs MMF | 1 | -0.455 | (-1.348, 0.439) | 0.00 | - | - | 0.319 |

Note: AZA, azathioprine; CH, chlorambucil; CON, non-immunosuppressive therapies (the control group); CsA, cyclosporine; CTX, cyclophosphamide; LEF, leflunomide; MMF, mycophenolate mofetil; RIT, rituximab; STE, steroids; TAC, tacrolimus.
