# Supplementary material for: Efficacy and safety of 12 immunosuppressive agents for idiopathic membranous nephropathy in adults: A pairwise and network meta-analysis
Source: Front Pharmacol. 2022 Jul 25;13:917532. doi: 10.3389/fphar.2022.917532 (PMC9358043; doi:10.3389/fphar.2022.917532)
Supplement: Supplementary file 7 [file DataSheet8.doc]

***Supplementary File 8: Result of sensitivity analysis***

1. **24 hours urine total protein**

**
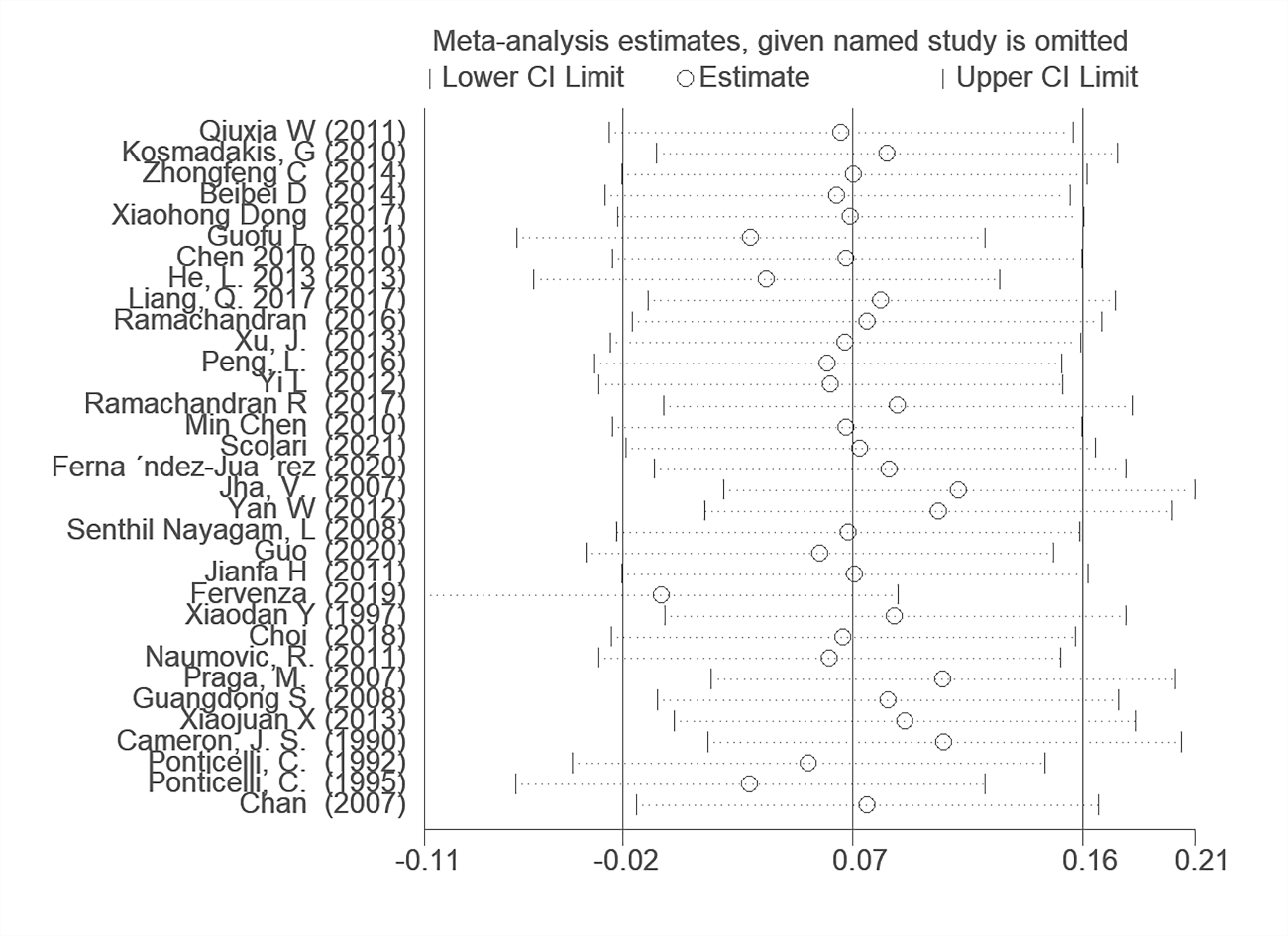
**

**2. Total remission**

**
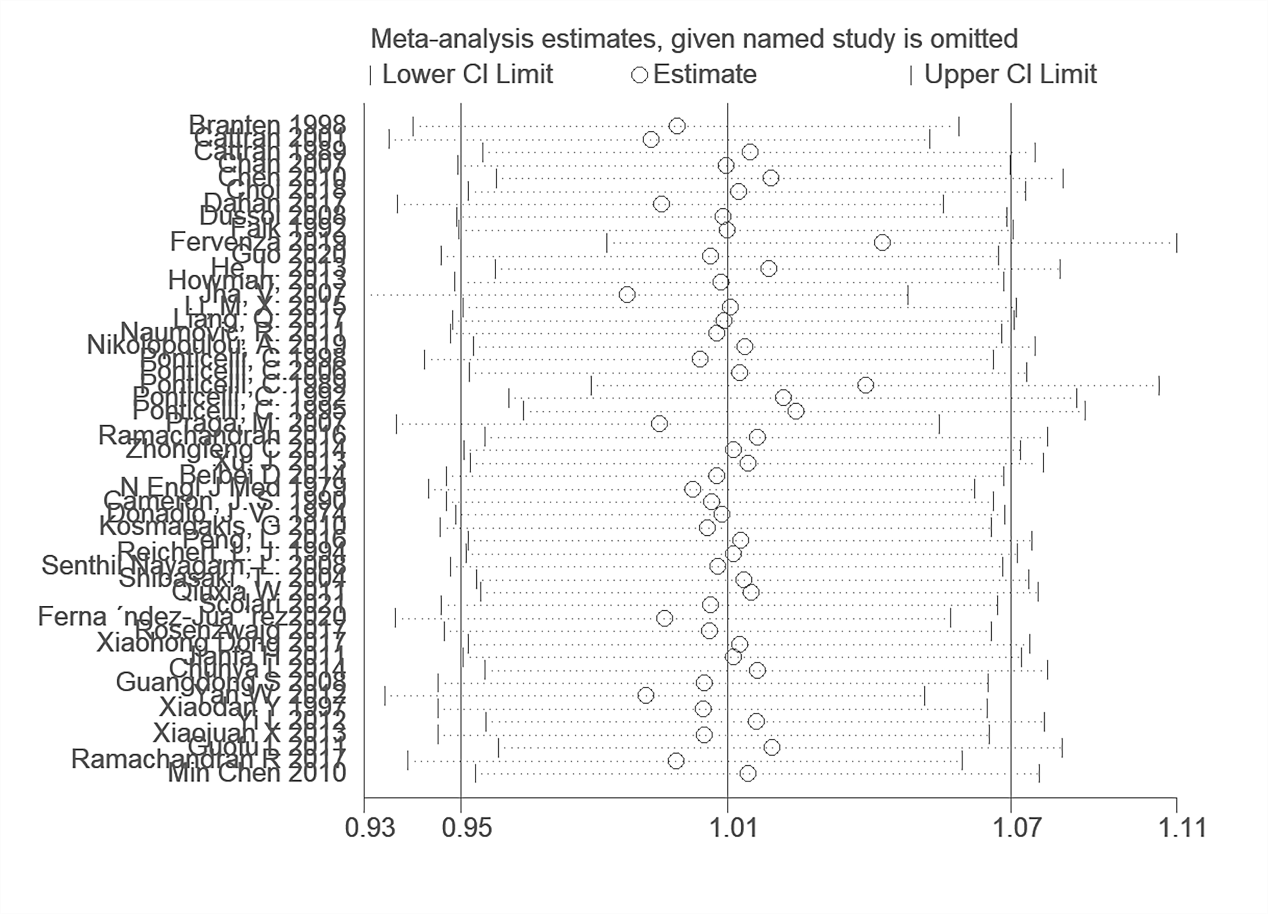
**
