# Supplementary material for: Efficacy and safety of 12 immunosuppressive agents for idiopathic membranous nephropathy in adults: A pairwise and network meta-analysis
Source: Front Pharmacol. 2022 Jul 25;13:917532. doi: 10.3389/fphar.2022.917532 (PMC9358043; doi:10.3389/fphar.2022.917532)
Supplement: Supplementary file 8 [file Table5.doc]

**eTable 4: The occurrence of adverse events**

| **Treatment** | **Total**  **Numbers** | **GS** | **HBP** | **Elevated ALT/AST** | **Infection** | **Leu** | **Anemia** | **Thr** | **GI or NDM** | **Tremble** | **MP** | **Dizziness and Headache** | **Alopecia** | **Herpes Zoster** | **Mal** | **Others** |
| --- | --- | --- | --- | --- | --- | --- | --- | --- | --- | --- | --- | --- | --- | --- | --- | --- |
| **CTX** | 464 | 61 (13.14%) | 9 (1.93%) | 56 (12.06%) | 116 (25.00%) | 70 (15.08%) | 20 (4.31%) | 4 (0.86%) | 33 (7.11%) | 3 (0.64%) | 10 (2.15%) | 5 (1.07%) | 20 (4.31%) | 4 (0.86%) | 3 (0.64%) | 50 (10.77%) |
| **CON** | 81 | 7 (8.64%) | 6 (7.40%) | 1 (1.23%) | 22 (27.16%) | 9 (11.11%) | 1 (1.23%) | 4 (4.93%) | 8 (9.87%) | 2 (2.46%) | 1 (1.23%) | 4 (4.93%) | 0 (0.00%) | 0 (0.00%) | 0 (0.00%) | 16 (19.75%) |
| **TAC** | 175 | 24 (13.71%) | 12 (6.85%) | 14 (8.00%) | 46 (26.28%) | 1 (0.57%) | 1 (0.57%) | 0 (0.00%) | 40 (22.85%) | 14 (8.00%) | 0 (0.00%) | 2 (1.14%) | 2 (1.14%) | 3 (1.71%) | 0 (0.00%) | 16 (9.14%) |
| **CsA** | 196 | 37 (18.87%) | 22 (11.22%) | 4 (2.04%) | 34 (17.34%) | 5 (2.55%) | 1 (0.51%) | 0 (0.00%) | 2 (1.02%) | 4 (2.04%) | 8 (4.08%) | 17 (8.67%) | 0 (0.00%) | 2 (1.02%) | 1 (0.51%) | 59 (30.10%) |
| **CH** | 98 | 11 (11.22%) | 0 (0.00%) | 2 (2.04%) | 14 (14.28%) | 42 (42.85%) | 1 (1.02%) | 1 (1.02%) | 9 (9.18%) | 2 (2.04%) | 0 (0.00%) | 3 (3.06%) | 0 (0.00%) | 7 (7.14%) | 0 (0.00%) | 6 (6.12%) |
| **MMF** | 56 | 14 (25.00%) | 1 (1.78%) | 2 (3.57%) | 21 (37.50%) | 2 (3.57%) | 3 (5.35%) | 0 (0.00%) | 2 (3.57%) | 0 (0.00%) | 4 (7.14%) | 0 (0.00%) | 1 (1.78%) | 1 (1.78%) | 2 (3.57%) | 3 (5.35%) |
| **RIT** | 169 | 23 (13.60%) | 6 (3.55%) | 0 (0.00%) | 50 (29.58%) | 2 (1.18%) | 9 (5.32%) | 0 (0.00%) | 1 (0.59%) | 7 (4.14%) | 17 (10.05%) | 13 (7.69%) | 0 (0.00%) | 1 (0.59%) | 3 (1.77%) | 37 (21.89%) |
| **STE** | 1 | 0 (0.00%) | 0 (0.00%) | 0 (0.00%) | 0 (0.00%) | 0 (0.00%) | 0 (0.00%) | 1 (100.00%) | 0 (0.00%) | 0 (0.00%) | 0 (0.00%) | 0 (0.00%) | 0 (0.00%) | 0 (0.00%) | 0 (0.00%) | 0 (0.00%) |
| **LEF** | 21 | 9 (42.85%) | 2 (9.52%) | 1 (4.76%) | 2 (9.52%) | 2 (9.52%) | 0 (0.00%) | 0 (0.00%) | 0 (0.00%) | 0 (0.00%) | 0 (0.00%) | 0 (0.00%) | 1 (4.76%) | 0 (0.00%) | 0 (0.00%) | 4 (19.04%) |
| **AZA** | 11 | 0 (0.00%) | 3 (27.27%) | 1 (9.09%) | 2 (18.18%) | 5 (45.45%) | 0 (0.00%) | 0 (0.00%) | 0 (0.00%) | 0 (0.00%) | 0 (0.00%) | 0 (0.00%) | 0 (0.00%) | 0 (0.00%) | 0 (0.00%) | 0 (0.00%) |
| **MIZ** | 11 | 0 (0.00%) | 0 (0.00%) | 0 (0.00%) | 1 (9.09%) | 0 (0.00%) | 0 (0.00%) | 0 (0.00%) | 0 (0.00%) | 0 (0.00%) | 0 (0.00%) | 0 (0.00%) | 0 (0.00%) | 1 (9.09%) | 0 (0.00%) | 9 (81.81%) |
| **ACTH** | 12 | 1 (8.33%) | 0 (0.00%) | 0 (0.00%) | 1 (8.33%) | 0 (0.00%) | 0 (0.00%) | 0 (0.00%) | 2 (16.66%) | 0 (0.00%) | 0 (0.00%) | 1 (8.33%) | 0 (0.00%) | 0 (0.00%) | 0 (0.00%) | 7 (58.33%) |
| **TAC+MMF** | 5 | 2 (40.00%) | 0 (0.00%) | 1 (20.00%) | 0 (0.00%) | 2 (40.00%) | 0 (0.00%) | 0 (0.00%) | 0 (0.00%) | 0 (0.00%) | 0 (0.00%) | 0 (0.00%) | 0 (0.00%) | 0 (0.00%) | 0 (0.00%) | 0 (0.00%) |

**Notes:** GS, Gastrointestinal symptoms; HBP, Hypertension; ALT, Alanine aminotransferase; AST, Aspartate aminotransferase; Leu, Leukopenia; Thr, Thrombotic episodes; GI, Glucose intolerance; NDM, New onset diabetes mellitus; MP, Muscular pain; Mal, Malignancy; ACTH, adrenocorticotropic hormone; AZA, azathioprine; CH, chlorambucil; CON, non-immunosuppressive therapies (the control group); CsA, cyclosporine; CTX, cyclophosphamide; LEF, leflunomide; MMF, mycophenolate mofetil; MZB, mizoribine; RIT, rituximab; STE, steroids; TAC, tacrolimus; TAC+MMF, tacrolimus combined mycophenolate mofetil.
