# Supplementary material for: Efficacy and safety of 12 immunosuppressive agents for idiopathic membranous nephropathy in adults: A pairwise and network meta-analysis
Source: Front Pharmacol. 2022 Jul 25;13:917532. doi: 10.3389/fphar.2022.917532 (PMC9358043; doi:10.3389/fphar.2022.917532)
Supplement: Supplementary file 9 [file Table2.docx]

| **eTable 2 Comparison between inconsistency model and consistency model** | | | | | |
| --- | --- | --- | --- | --- | --- |
| **Outcomes** | **Dbar** | **Data points** | **Pd** | **DIC** | **I2** |
| Total Remission (Consistency model) | 106.44 | 104 | 77.10 | 183.55 | 3% |
| Total Remission (Inconsistency model) | 109.61 | 104 | 77.48 | 187.10 | 6% |
| 24h UTP (Consistency model) | 65.56 | 65 | 60.06 | 125.63 | 2% |
| 24h UTP (Inconsistency model) | 63.74 | 65 | 60.62 | 124.36 | 0% |
