# Supplementary material for: Efficacy and safety of 12 immunosuppressive agents for idiopathic membranous nephropathy in adults: A pairwise and network meta-analysis
Source: Front Pharmacol. 2022 Jul 25;13:917532. doi: 10.3389/fphar.2022.917532 (PMC9358043; doi:10.3389/fphar.2022.917532)
Supplement: Supplementary file 10 [file Table3.docx]

| **Table S3 Characteristics of the included studies.** | | | | | | | | | |
| --- | --- | --- | --- | --- | --- | --- | --- | --- | --- |
| Study | County | Sex(M/F) | Sample size(T/C) | Mean age  (Year, T/C) | Study duration(mo) | Follow-up  (mo, T/C) | Interventions  (T/C) | Outcomes | Adverse |
| Branten AJ 1998 [16] | Netherlands | 30/2 | 17/15 | 51/53 | 12 | 26/38 | CTX/CH | TR | Respiratory tract infection+ Leukopenia |
| Cattran DC 2001 [17] | American | 42/9 | 28/23 | 49/47 | 6 | 18/18 | CsA/CON | TR | NA |
| Cattran DC 1989 [18] | Canada | 105/53 | 81/77 | 45/46 | 6 | 48/48 | STE/CON | TR | NA |
| Chan TM 2007 [19] | China | 13/7 | 11/9 | 49.5/49.5 | 6 | 15/15 | MMF/CON | TR + 24h UTP | Gastrointestinal symptoms  +respiratory tract infection |
| Chen M 2010 [20] | China | 41/32 | 39/34 | 47.2/48.6 | 12 | 12/12 | TAC/CTX | TR + 24h UTP | Glucose intolerance+ infection |
| Choi JY 2018 [21] | Korea | 25/14 | 21/18 | 57.7/52.7 | 12 | 12/12 | MMF/CsA | TR + 24h UTP | Infections + Gastrointestinal symptoms |
| Dahan K 2017 [22] | France | 52/23 | 37/38 | 53/58.5 | 6 | 24/24 | RIT/CON | TR | NA |
| Dussol B 2008 [23] | France | 32/4 | 19/17 | 55.9/47.8 | 12 | 12/12 | MMF/CON | TR | Infection +Muscular pain |
| Falk RJ 1992 [24] | American | 16/10 | 13/13 | 43.3/46 | 24 | 31.9/26.5 | CTX/CON | TR | NA |
| Fervenza FC 2019 [25] | American | 100/30 | 65/65 | 51.9/52.2 | 12 | 24/24 | RIT/CsA | TR + 24h UTP | Respiratory tract infection +Nausea or vomiting |
| Guo Y 2020 [26] | China | 32/28 | 30/30 | 49.13/49.20 | 6 | 6/6 | LEF/CTX | TR + 24h UTP | Alopecia + Vomiting |
| He L 2013 [27] | China | 39/17 | 28/28 | 45.4/47.2 | 12 | 12/12 | TAC/CTX | TR + 24h UTP | Gastrointestinal syndrome+ Gouty arthritis |
| Howman A 2013 [28] | Britain | NA | 33/36/37 | 58/58/56 | 12 | 36/36 | CH/CsA/CON | TR | Leucopenia+ Hypertension +Infection |
| Jha V 2007 [29] | India | 57/36 | 47/46 | 38.0/37.2 | 6 | 120/120 | CTX/CON | TR + 24h UTP | Infection |
| Li MX 2015 [30] | China | 20/7 | 14/13 | 75.1/74.8 | 12 | 48/48 | CsA/STE | TR | NA |
| Liang Q 2017 [31] | China | 25/33 | 30/28 | 48.2/53.9 | 12 | 22/22 | TAC/CTX | TR + 24h UTP | Glucose intolerance+  Abnormal aminotransferase + Gastrointestinal symptoms |
| Naumovic R 2011 [32] | Serbia | 19/4 | 10/13 | 39.2/47.5 | 36 | 36/36 | CsA/AZA | TR + 24h UTP | Nausea+ Leucopenia |
| Nikolopoulou A 2019 [33] | Britain | 24/16 | 20/20 | 55/48 | 12 | 18/18 | TAC/TAC+MMF | TR | vomiting + Urinary tract infection |
| Ponticelli C 1998 [34] | Italy | 66/21 | 44/43 | 50/48 | 12 | 36/42 | CH/CTX | TR | NA |
| Ponticelli C 2006 [35] | Italy | 19/13 | 16/16 | 51.4/48 | 12 | 36/36 | CTX/ACTH | TR | Glucose intolerance +Dizziness |
| Ponticelli C 1989 [36] | Italy | 63/18 | 42/39 | 43.5/42 | 6 | 24/24 | CH/CON | TR + 24h UTP | Peptic ulcers +Gastric intolerance |
| Ponticelli C 1992 [37] | Italy | 59/33 | 45/47 | 46/47 | 6 | 48/48 | CH/STE | TR + 24h UTP | Leukopenia+ Fever+ Gastric discomfort |
| Ponticelli C 1995 [38] | Italy | NA | 42/39 | NA | 6 | 120/120 | CH/CON | TR | Peptic ulcer +Leukopenia +tremor |
| Praga M 2007 [39] | Spain | 40/8 | 25/23 | 43.7/50.1 | 18 | 30/30 | TAC/CON | TR + 24h UTP | Glucose intolerance +Diarrhea+ Chest pain |
| Ramachandran R 2016 [40] | India | 47/23 | 35/35 | 38.6/40.8 | 12 | 12/12 | TAC/CTX | TR + 24h UTP | Infections+ Diabetes mellitus+ Gastrointestinal symptoms |
| Xu J 2013 [41] | China | 61/23 | 49/35 | 56.3/57.8 | 6 | 18/18 | TAC/CTX | TR + 24h UTP | Infection+ Glucose intolerance |
| N Engl J Med 1979 [42] | American | 42/30 | 34/38 | 44/42 | 6 | 24/24 | STE/CON | TR | NA |
| Cameron JS 1990 [43] | Britain | 86/17 | 52/51 | 45/44 | 12 | 36/36 | STE/CON | TR + 24h UTP | NA |
| Donadio JV 1974 [44] | American | 17/5 | 11/11 | 42.1/46.6 | 12 | 24/24 | CTX/CON | TR | Alopecia+ Nausea |
| Kosmadakis G 2010 [45] | Greece | 12/6 | 8/10 | 55.4/50.5 | 9 | 9/9 | CTX/CsA | TR + 24h UTP | NA |
| Peng L 2016 [46] | China | 47/43 | 29/28/29 | 43.9/40.8/39.9 | 9 | 9/9 | TAC/CTX/MMF | TR + 24h UTP | Infection +Hand tremor+ Gastrointestinal reaction |
| Reichert LJ 1994 [47] | Netherlands | 17/1 | 9/9 | 49/45 | 6 | 36/36 | CTX/CH | TR | Infection+ Leukopenia |
| Senthil Nayagam L 2008 [48] | India | 16/5 | 11/10 | 30.2/33.1 | 6 | 18/16 | MMF/CTX | TR + 24h UTP | NA |
| Shibasaki T 2004 [49] | Japan | 10/7 | 11/6 | 45/48 | 6 | 24/24 | MIZ/CON | TR | Hyperuricemia +Pneumonia and rash |
| Scolari F 2021 [50] | Italy | 53/21 | 37/37 | 54/55 | 12 | 36/36 | RIT/CTX | TR | Infectious +Drug infusion reaction/Drug intolerance |
| Fernández-Juárez G 2021 [51] | Spain | 55/31 | 43/43 | 55.2/56.2 | 6 | 24/24 | RIT/CTX | TR + 24h UTP | Gastrointestinal +Infections |
| Rosenzwajg M 2017 [52] | France | 20/5 | 16/9 | 57/49 | 6 | 6/6 | RIT/CON | TR | NA |
| Qiuxia W 2011 [53] | China | 27/9 | 18/18 | 36.2/36.2 | 6 | 12/12 | CTX/CsA | TR + 24h UTP | Tremble +Elevated liver enzymes |
| Chunya L 2014 [54] | China | 40/28 | 34/34 | 41.6/42.2 | 12 | 12/12 | MMF/CTX | TR | Leukopenia+ Gastrointestinal symptoms |
| Guangdong S 2008 [55] | China | 14/6 | 10/10 | 49.54/49.54 | 6 | 6/6 | TAC/LEF | TR + 24h UTP | Hypertension+ Diarrhea |
| Yan W 2012 [56] | China | 45/14 | 29/30 | 45.41/44.97 | 6 | 6/6 | CTX/CON | TR + 24h UTP | Gastrointestinal symptoms+ Elevated liver enzymes |
| Xiaodan Y 1997 [57] | China | 13/10 | 13/10 | 36.1/39.5 | 12 | 15/15 | CsA/CON | TR + 24h UTP | Gastrointestinal symptoms |
| Xiaojuan X 2013 [58] | China | 20/10 | 15/15 | NA | 6 | NA | TAC/LEF | TR + 24h UTP | Gastrointestinal symptoms +leukopenia |
| Zhongfeng C 2014 [59] | China | 27/13 | 20/20 | 51.5/51.5 | 12 | 12/12 | CTX/CsA | TR + 24h UTP | Leukopenia + Hypertension +  Abnormal aminotransferase |
| Beibei D 2014 [60] | China | 28/14 | 21/21 | 37.8/36.6 | 12 | 12/12 | CTX/CsA | TR + 24h UTP | Gastrointestinal symptoms  +Abnormal aminotransferase |
| Xiaohong D 2017 [61] | China | 29/17 | 23/23 | 35.9/35.8 | 12 | 12/12 | CTX/CsA | TR + 24h UTP | Leukopenia+ Hypertension+  Abnormal aminotransferase |
| Jianfa H 2011 [62] | China | 31/21 | 26/26 | 50±14 | 6 | 6/6 | CTX/LEF | TR + 24h UTP | Gastrointestinal symptoms +Alopecia |
| Guofu L 2011 [63] | China | 49/27 | 36/40 | 44.8/45.2 | 12 | 12/12 | CTX/CsA | TR + 24h UTP | Leukopenia + Abnormal aminotransferase |
| Yi L 2012 [64] | China | NA | 15/15 | 53.2±10.8 | 6 | 6/6 | TAC/CTX | TR + 24h UTP | Gastrointestinal symptoms + Alopecia |
| Ramachandran R 2017 [65] | India | NA | 35/35 | NA | 12 | 24/24 | TAC/CTX | TR + 24h UTP | Infection + Abnormal aminotransferase |
| Jurubita R 2012 [66] | Romania | NA | 9/9 | NA | 12 | 12/12 | MMF/CsA | TR | Hypertension+ Hypertrichosis + Gingival hypertrophy |

**Abbreviations:** mo, month; M, male; F, female; T, treatment group; C, control group; ACTH, adrenocorticotropic hormone; AZA, azathioprine; CH, chlorambucil; CON, non-immunosuppressive therapies (the control group); CsA, cyclosporine; CTX, cyclophosphamide; LEF, leflunomide; MMF, mycophenolate mofetil; MZB, mizoribine; RIT, rituximab; STE, steroids; TAC, tacrolimus; TAC+MMF, tacrolimus combined mycophenolate mofetil; TR, Total remission; 24h UTP, 24 hours urine total protein; NA, not available.
