# Supplementary material for: Efficacy and safety of 12 immunosuppressive agents for idiopathic membranous nephropathy in adults: A pairwise and network meta-analysis
Source: Front Pharmacol. 2022 Jul 25;13:917532. doi: 10.3389/fphar.2022.917532 (PMC9358043; doi:10.3389/fphar.2022.917532)
Supplement: Supplementary file 11 [file DataSheet9.doc]

***Supplementary File 9: General classification of drugs***


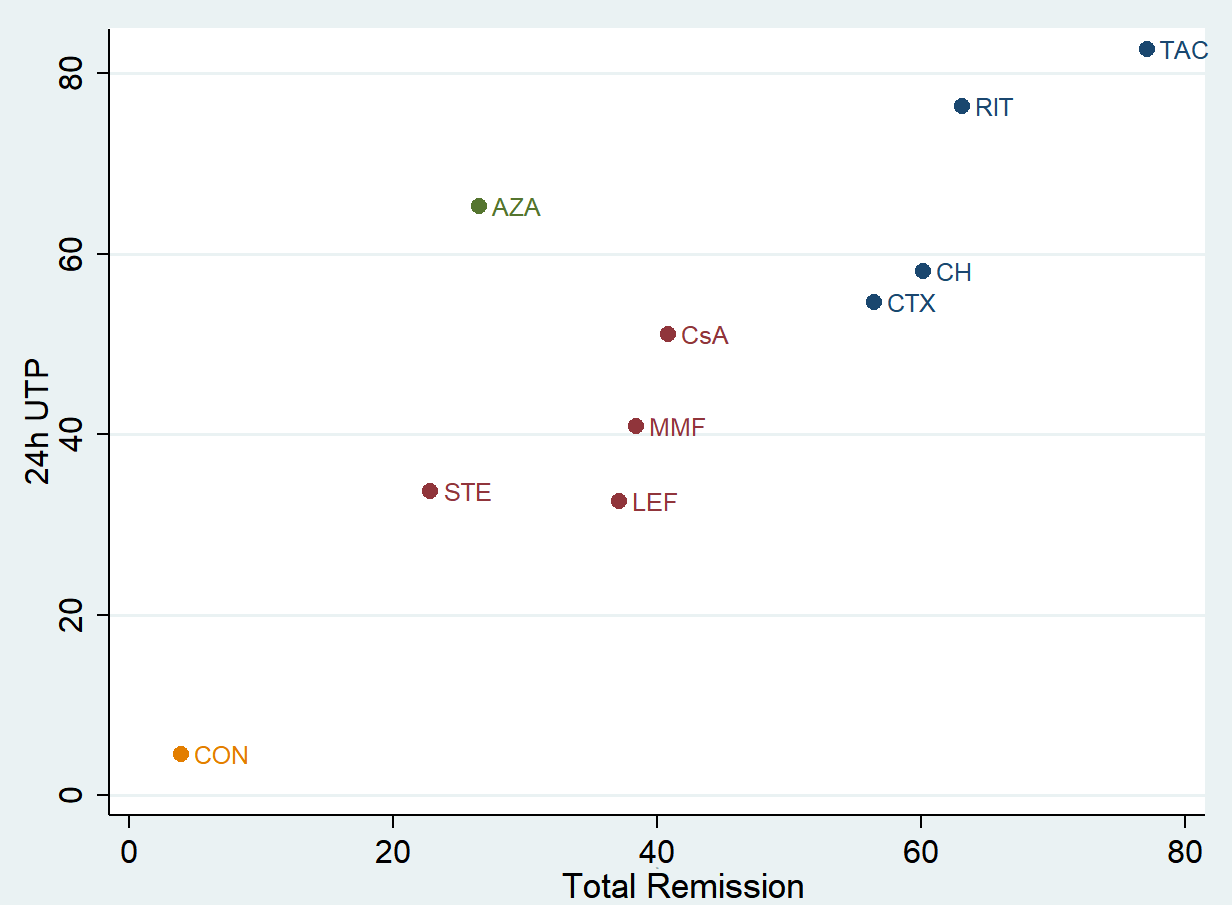


**eFig.5** Horizontal axis represents the SUCRA of different treatments on total remission, whereas Longitudinal axis represents the SUCRA of different treatments on 24h UTP. AZA, azathioprine; CH, chlorambucil; CON, non-immunosuppressive therapies (the control group); CsA, cyclosporine; CTX, cyclophosphamide; LEF, leflunomide; MMF, mycophenolate mofetil; RIT, rituximab; STE, steroids; TAC, tacrolimus. Significant effect group contains TAC, RIT; Moderate effect group consists of CTX, MMF and CH; Low effect group includes LEF, CsA, AZA and STE; Very low effect group means CON.
