# Supplementary material for: Efficacy and safety of 12 immunosuppressive agents for idiopathic membranous nephropathy in adults: A pairwise and network meta-analysis
Source: Front Pharmacol. 2022 Jul 25;13:917532. doi: 10.3389/fphar.2022.917532 (PMC9358043; doi:10.3389/fphar.2022.917532)
Supplement: Supplementary file 12 [file DataSheet2.docx]

***Supplementary File2: Risk of bias table for included studies***

eTable1. Quality evaluation of enrolled RCTs according to Cochrane Handbook.

| **Study** | **Random sequence generation** | **Random concealment** | **Blinding of participants and personnel** | **Blinding of outcome assessment** | **Incomplete outcome data** | **Selective reporting** | **Other bias** |
| --- | --- | --- | --- | --- | --- | --- | --- |
| Branten AJ 1998 [16] | The trial only reported “randomized”. | patients were asked to participate in a randomized trial | No information was provided. | No information was provided. | The trial did not report any dropouts. | Results of all primary outcomes were reported. | Unclear |
| Cattran DC 2001 [17] | Randomization was performed by the clinical coordinating center from a table of random numbers | Randomization was stratified by center in blocks of two to ensure a balance between groups. | The patients were masked in regards to active versus placebo assignment, but the physicians were not for safety reasons and because the end points were objective and measured centrally by a lab blinded to patient designation. | No information was provided. | The trial did not report any dropouts. | Results of all primary outcomes were reported | Unclear |
| Cattran DC 1989 [18] | Random numbers | the patient was assigned by the trial coordinator according to a table of random numbers | Neither the patient nor the physician was blinded to the patient's assignment | No information was provided. | The trial did not report any dropouts. | Results of all primary outcomes were reported. | Unclear |
| Chan TM 2007 [19] | The trial only reported “randomized”. | No information was provided. | No information was provided. | No information was provided. | The trial did not report any dropouts. | Results of all primary outcomes were reported. | Unclear |
| Chen M 2010 [20] | Randomization was per-formed by a clinical coordinating center using a table of random numbers and was stratified by centers. | Allocation concealment was performed by enclosing assignments in sequentially numbered, opaque-closed envelopes | No information was provided. | No information was provided. | The trial did not report any dropouts. | Results of all primary outcomes were reported | Unclear |
| Choi JY 2018 [21] | The table of random numbers was generated using the SAS randomization program. | Allocation concealment was done by sealed sequentially numbered opaque envelopes. They were consecutively numbered and bottles were provided to the patients according to the number allocated | No information was provided. | No information was provided. | The trial did not report any dropouts. | Results of all primary outcomes were reported | Unclear |
| Dahan K 2017 [22] | The trial only reports “randomized”. | No information was provided. | The trial was not blinded. | Data were collected in a paper case report form and entered into a database. Data assessors were blinded to treatment allocation. | The trial did not report any dropouts. | Results of all primary outcomes were reported. | Unclear |
| Dussol B 2008 [23] | Patients were randomly assigned to either a control group or a treatment group | Randomization was stratified according to sex and center. | No information was provided. | No information was provided. | The trial did not report any dropouts. | Results of all primary outcomes were reported. | Unclear |
| Falk RJ 1992 [24] | Patients were randomly assigned to one of two treatments | All patients were randomized under the same computergenerated randomization table through the central Glomerular Disease Collaborative Network office. | No information was provided. | No information was provided. | The trial did not report any dropouts. | Results of all primary outcomes were reported. | Unclear |
| Fervenza FC 2019 [25] | This was an open-label, investigator-initiated trial. | No information was provided. | No information was pro-vided. | No information was provided. | The trial did not report any dropouts. | Results of all primary outcomes were reported. | Unclear |
| Guo Y 2020 [26] | The patients were randomly divided into an experimental group or a control group | The patients were divided randomly using a random numerical table method | No information was pro-vided. | No information was provided. | The trial did not report any dropouts. | Results of all primary outcomes were reported. | Unclear |
| He L 2013 [27] | The randomization was performed through a preprinted randomization table. | No information was provided. | No information was provided. | No information was provided. | The trial did not report any dropouts. | Results of all primary outcomes were reported. | Unclear |
| Howman A 2013 [28] | Eligible patients were randomly assigned by a member of staff | A random numbers table had been prepared to allocate patients to one of three groups | Treatment allocation was communicated by fax to the clinician entering the patient into the trial. We did not attempt to mask patients or investigators. | No information was provided. | The trial did not report any dropouts. | Results of all primary outcomes were reported. | Unclear |
| Jha V 2007 [29] | This was an open-label, investigator-initiated trial. | No information was provided. | No information was provided. | No information was provided. | The trial did not report any dropouts. | Results of all primary outcomes were reported. | Unclear |
| Li MX 2015 [30] | The trial only reported randomized” | No information was provided. | No information was provided. | No information was provided. | The trial did not report any dropouts. | Results of all primary outcomes were reported. | Unclear |
| Liang Q 2017 [31] | A few patients chose the regimen. | No information was provided. | No information was provided. | No information was provided. | The trial did not report any dropouts. | Results of all primary outcomes were reported. | Unclear |
| Naumovic R 2011 [32] | Patients were randomly assigned to receive either cyclosporine or azathioprine for 6 months | No information was provided. | No information was provided. | No information was provided. | The trial did not report any dropouts. | Results of all primary outcomes were reported. | Unclear |
| Nikolopoulou A 2019 [33] | Eligible patients were randomly assigned to receive either tacrolimus or tacrolimus in combination with mycophenolate mofetil. | Allocation concealment was performed by enclosing assignments in sequentially numbered, opaque, sealed envelopes. | No information was provided. | Data were collected in paper case report forms and progress of the trial was reported to and monitored by the Research Group. | The trial did not report any dropouts. | Results of all primary outcomes were reported. | Unclear |
| Ponticelli C 1998 [34] | Patiens were assigned consecutively to one of two treatment regimens, according to a center-stratified random order. | A center-stratified random order. | No information was provided. | No information was provided. | The trial did not report any dropouts. | Results of all primary outcomes were reported. | Unclear |
| Ponticelli C 2006 [35] | The coordinating center assigned patients consecutively by telephone to 1 of the 2 treatment regimens in a centralized randomized order. | With assignation produced by a table from a statistical textbook. | The sequence was concealed until intervention was assigned | Data were collected by the local pathologist and sent at the end of the study to the coordinating center | The trial did not report any dropouts. | Results of all primary outcomes were reported. | Unclear |
| Ponticelli C 1989 [36] | Each patient was randomly assigned to either specific or supportive therapy. | The indications for therapy were contained in sealed, completely opaque envelopes numbered in sequence according to a table of random numbers. | The study should be an open. rather than a blinded, randomized trial. | No information was provided. | The trial did not report any dropouts. | Results of all primary outcomes were reported. | Unclear |
| Ponticelli C 1992 [37] | Patiens were assigned consecutively to one of two treatment regimens, according to a random order. | No information was provided. | No information was provided. | No information was provided. | The trial did not report any dropouts. | Results of all primary outcomes were reported. | Unclear |
| Ponticelli C 1995 [38] | Patients were randomly assigned to one of two treatment groups. | No information was provided. | No information was provided. | No information was provided. | The trial did not report any dropouts. | Results of all primary outcomes were reported. | Unclear |
| Praga M 2007 [39] | The trial only reported “randomized”. | No information was provided. | No information was provided. | No information was provided. | The trial did not report any dropouts. | Results of all primary outcomes were reported. | Unclear |
| Ramachandran R 2016 [40] | Participants were ran-domly assigned following computer-based random numbers to one of the two treatment groups. | The author who performed the randomization did not participate in the enrolment and allocation of treatment to the participants and concealment was done using sequentially labeled sealed envelopes containing the specified intervention. | Both the patients, treating physicians, and individuals were not blinded to patient treatment assignments. | Both the patients, treating physicians, and individuals assessing clinical outcomes and analyzing data. | The trial did not report any dropouts. | Results of all primary outcomes were reported. | Unclear |
| Xu J 2013 [41] | The trial only reputed randomized”. | No information was provided. | No information was provided. | No information was provided. | The trial did not report any dropouts. | Results of all primary outcomes were reported. | Unclear |
| N Engl J Med 1979 [42] | Patients were randomly allocated to prednisone or placebo. | Randomization was stratified according to initial histologic diagnosis with the light microscope | No information was provided. | No information was provided. | The trial did not report any dropouts. | Results of all primary outcomes were reported. | Unclear |
| Cameron JS 1990 [43] | Randomization was performed centrally, and coded tablets given locally from bottles supplied from the coordinator | Randomization was stratified for sex, but not for MHC tissue type or other features. | No information was provided. | No information was provided. | The trial did not report any dropouts. | Results of all primary outcomes were reported. | Unclear |
| Donadio JV 1974 [44] | The patient was placed in one of two treatment groups by random assignment | A table of random numbers | Neither patient nor clinician knew what treatment was going to be given | No information was provided. | The trial did not report any dropouts. | Results of all primary outcomes were reported. | Unclear |
| Kosmadakis G 2010 [45] | The trial only reported “randomized” | The person doing the randomization was blinded. | The recruiting and treating doctors, as well as the patients, were not blinded on the type of treatment throughout the medication period. | The trial did not report any dropouts. | The trial did not report any dropouts. | Results of all primary outcomes were reported. | Unclear |
| Peng L 2016 [46] | The trial only reported “randomized” | No information was provided. | No information was provided. | No information was provided. | The trial did not report any dropouts. | Results of all primary outcomes were reported. | Unclear |
| Reichert LJ 1994 [47] | Patients were randomly assigned to one of two treatment protocols | No information was provided. | No information was provided. | No information was provided. | The trial did not report any dropouts. | Results of all primary outcomes were reported. | Unclear |
| Senthil Nayagam L 2008 [48] | The trial only reported “randomized” | No information was provided. | No information was provided. | No information was provided. | The trial did not report any dropouts. | Results of all primary outcomes were reported. | Unclear |
| Shibasaki T 2004 [49] | Patients were randomly assigned to one of two treatment groups | No information was provided. | Neither the patients nor their physicians were masked to the assigned treatments | No information was provided. | The trial did not report any dropouts. | Results of all primary outcomes were reported. | Unclear |
| Scolari F 2021 [50] | An analyst from a distant site, with no clinical involvement in the trial, generated the randomization lists | An analyst from a distant site, with no clinical involvement in the trial, kept randomization lists concealed | No information was provided | No information was provided | The trial did not report any dropouts. | Results of all primary outcomes were reported. | Unclear |
| Fernández-Juárez G 2021 [51] | The trial used a random number–producing algorithm in central computer systems for simple randomization. | The subject numbers were assigned sequentially as each subject entered the study. | No information was provided. | No information was provided. | The trial did not report any dropouts. | Results of all primary outcomes were reported. | Unclear |
| Rosenzwajg M 2017 [52] | The trial only reported “randomized” | No information was provided. | No information was provided. | No information was provided. | The trial did not report any dropouts. | Results of all primary outcomes were reported. | Unclear |
| Qiuxia W 2011 [53] | The trial only reported “randomized” | No information was provided. | No information was provided. | No information was provided. | The trial did not report any dropouts. | Results of all primary outcomes were reported. | Unclear |
| Chunya L 2014 [54] | The trial only reported “randomized” | No information was provided. | No information was provided. | No information was provided. | The trial did not report any dropouts. | Results of all primary outcomes were reported. | Unclear |
| Guangdong S 2008 [55] | The trial only reported “randomized” | No information was provided. | No information was provided. | No information was provided. | The trial did not report any dropouts. | Results of all primary outcomes were reported. | Unclear |
| Yan W 2012 [56] | Patients were randomly assigned to one of two treatment groups | By means of random number table | No information was provided. | No information was provided. | The trial did not report any dropouts. | Results of all primary outcomes were reported. | Unclear |
| Xiaodan Y 1997 [57] | The trial only reported “randomized” | No information was provided. | No information was provided. | No information was provided. | The trial did not report any dropouts. | Results of all primary outcomes were reported. | Unclear |
| Xiaojuan X 2013 [58] | The trial only reported “randomized” | No information was provided. | No information was provided. | No information was provided. | The trial did not report any dropouts. | Results of all primary outcomes were reported. | Unclear |
| Zhongfeng C 2014 [59] | Patients were randomly assigned to one of two treatment groups | No information was provided. | No information was provided. | No information was provided. | The trial did not report any dropouts. | Results of all primary outcomes were reported. | Unclear |
| Beibei D 2014 [60] | Patients were randomly assigned to one of two treatment groups | By means of random number table | No information was provided. | No information was provided. | The trial did not report any dropouts. | Results of all primary outcomes were reported. | Unclear |
| Xiaohong D 2017 [61] | Patients were randomly assigned to one of two treatment groups | By means of random number table | No information was provided. | No information was provided. | The trial did not report any dropouts. | Results of all primary outcomes were reported. | Unclear |
| Jianfa H 2011 [62] | Patients were randomly assigned to one of two treatment groups | No information was provided. | No information was provided. | No information was provided. | The trial did not report any dropouts. | Results of all primary outcomes were reported. | Unclear |
| Guofu L 2011 [63] | Patients were randomly assigned to one of two treatment groups | No information was provided. | No information was provided. | No information was provided. | The trial did not report any dropouts. | Results of all primary outcomes were reported. | Unclear |
| Yi L 2012 [64] | Patients were randomly assigned to one of two treatment groups | No information was provided. | No information was provided. | No information was provided. | The trial did not report any dropouts. | Results of all primary outcomes were reported. | Unclear |
|  |  |  |  |  |  |  |  |
| Ramachandran R 2017 [65] | Patients were randomly assigned to one of two treatment groups | No information was provided. | No information was provided. | No information was provided. | The trial did not report any dropouts. | Results of all primary outcomes were reported. | Unclear |
| Jurubita R 2012  [66] | Patients were randomly assigned to one of two treatment groups | No information was provided. | No information was provided. | No information was provided. | The trial did not report any dropouts. | Results of all primary outcomes were reported. | Unclear |


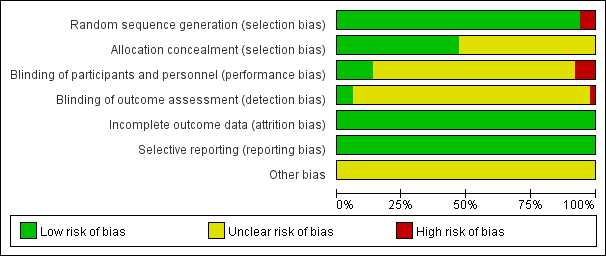


eFig.1 Risk of bias graph


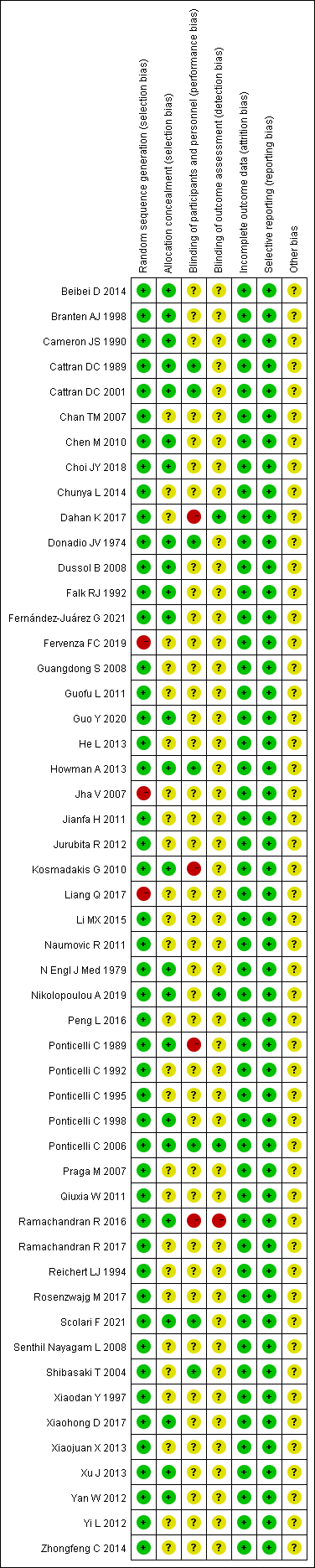


eFig.2 Risk of bias summary
