# Supplementary material for: Efficacy and safety of 12 immunosuppressive agents for idiopathic membranous nephropathy in adults: A pairwise and network meta-analysis
Source: Front Pharmacol. 2022 Jul 25;13:917532. doi: 10.3389/fphar.2022.917532 (PMC9358043; doi:10.3389/fphar.2022.917532)
Supplement: Supplementary file 13 [file DataSheet6.doc]

***Supplementary File 6: Evaluation of meta-regression***

**eTable5. Results of meta-regression for TR**

| **Variables** | **Comparison** | **Regression coefficient** | **95%CI** |
| --- | --- | --- | --- |
| Age | CTX vs CON | 0.826 | (-0.534, 2.251) |
|  | AZA vs CON | 0.138 | (-24.229, 30.202) |
|  | MIZ vs CON | 0.543 | (-29.394, 31.719) |
|  | ACTH vs CON | -0.056 | (-23.593, 22.949) |
|  | TAC+MMF vs CON | 0.943 | (-15.692, 25.705) |
|  | CsA vs CON | -1.069 | (-2.396, 0.242) |
|  | TAC vs CON | 0.603 | (-1.397,2.724) |
|  | RIT vs CON | -1.977 | (-8.010, 2.831) |
|  | STE vs CON | -0.425 | (-2.030, 1.166) |
|  | CH vs CON | -0.758 | (-2.713, 1.147) |
|  | MMF vs CON | -0.182 | (-2.286, 1.953) |
|  | LEF vs CON | -2.364 | (-46.481, 20.500) |
| Study duration | CTX vs CON | -0.665 | (-1.933, 0.597) |
|  | AZA vs CON | 1.064 | (-13.181, 26.338) |
|  | MIZ vs CON | 1.076 | (-15.416, 23.272) |
|  | ACTH vs CON | 1.535 | (-20.852, 36.099) |
|  | TAC+MMF vs CON | -0.797 | (-30.664, 25.100) |
|  | CsA vs CON | 0.602 | (-2.792, 1.509) |
|  | TAC vs CON | -0.929 | (-2.868, 0.922) |
|  | RIT vs CON | 1.356 | (-1.228, 4.116) |
|  | STE vs CON | 0.402 | (-2.198, 3.126) |
|  | CH vs CON | -3.551 | ***(-6.946, -0.443)*** |
|  | MMF vs CON | 0.904 | (-1.968, 4.053) |
|  | LEF vs CON | -0.604 | (-20.776, 14.514) |
| Sample Size | CTX vs CON | 0.658 | (-0.723, 2.066) |
|  | AZA vs CON | 1.274 | (-15.868, 26.455) |
|  | MIZ vs CON | 4.130 | (-19.533, 87.980) |
|  | ACTH vs CON | 0.565 | (-21.325, 27.397) |
|  | TAC+MMF vs CON | -0.884 | (-52.603, 35.454) |
|  | CsA vs CON | -0.301 | (-1.918, 1.344) |
|  | TAC vs CON | -0.640 | (-2.979, 1.567) |
|  | RIT vs CON | 0.914 | (-1.268, 3.079) |
|  | STE vs CON | -0.770 | (-1.930, 0.406) |
|  | CH vs CON | 1.091 | (-0.893, 3.174) |
|  | MMF vs CON | 2.112 | (-0.547, 5.017) |
|  | LEF vs CON | 0.596 | (-3.136, 4.701) |

Abbreviation: ACTH, adrenocorticotropic hormone; AZA, azathioprine; CH, chlorambucil; CON, non-immunosuppressive therapies (the control group); CsA, cyclosporine; CTX, cyclophosphamide; LEF, leflunomide; MMF, mycophenolate mofetil; MZB, mizoribine; RIT, rituximab; STE, steroids; TAC, tacrolimus; TAC+MMF, tacrolimus combined mycophenolate mofetil.

**eTable6. Results of meta-regression for 24h UTP**

| **Variables** | **Comparison** | **Regression coefficient** | **95%CI** |
| --- | --- | --- | --- |
| Age | CTX vs CON | -0.373 | (-2.666, 1.898) |
|  | AZA vs CON | -0.099 | (-28.989, 27.749) |
|  | CsA vs CON | 0.638 | (-1.719, 3.042) |
|  | TAC vs CON | -0.142 | (-3.044, 2.789) |
|  | RIT vs CON | 0.418 | (-5.342, 6.319) |
|  | STE vs CON | -1.584 | (-20.390, 12.528) |
|  | CH vs CON | 2.066 | (-5.253, 11.594) |
|  | MMF vs CON | 0.994 | (-1.717, 3.665) |
|  | LEF vs CON | 2.907 | (-16.958, 32.665) |
| Study duration | CTX vs CON | -0.444 | (-2.951, 2.071) |
|  | AZA vs CON | -0.247 | (-17.146, 15.445) |
|  | CsA vs CON | -0.911 | (-5.031, 3.018) |
|  | TAC vs CON | 0.824 | (-1.399, 3.300) |
|  | RIT vs CON | -1.604 | (-6.165, 2.910) |
|  | STE vs CON | 1.886 | (-3.223, 7.863) |
|  | CH vs CON | 0.488 | (-13.972, 17.564) |
|  | MMF vs CON | -2.878 | (-9.404, 2.417) |
|  | LEF vs CON | 4.705 | (-22.309, 62.133) |
| Sample size | CTX vs CON | -0.033 | (-1.851, 1.762) |
|  | AZA vs CON | 1.644 | (-19.443, 34.000) |
|  | CsA vs CON | -2.063 | (-4.875, 0.526) |
|  | TAC vs CON | 0.865 | (-1.483, 3.281) |
|  | RIT vs CON | -4.149 | (-9.008, 0.197) |
|  | STE vs CON | 2.572 | (-6.424, 19.566) |
|  | CH vs CON | 2.325 | (-5.899, 12.511) |
|  | MMF vs CON | -1.454 | (-4.505, 1.529) |
|  | LEF vs CON | -3.390 | (-7.510, 0.543) |

Abbreviation: CH, chlorambucil; CON, non-immunosuppressive therapies (the control group); CsA, cyclosporine; CTX, cyclophosphamide; LEF, leflunomide; MMF, mycophenolate mofetil; MZB, mizoribine; RIT, rituximab; STE, steroids; TAC, tacrolimus.
